# Mining Host-Pathogen Protein Interactions to Characterize *Burkholderia mallei* Infectivity Mechanisms

**DOI:** 10.1371/journal.pcbi.1004088

**Published:** 2015-03-04

**Authors:** Vesna Memišević, Nela Zavaljevski, Seesandra V. Rajagopala, Keehwan Kwon, Rembert Pieper, David DeShazer, Jaques Reifman, Anders Wallqvist

**Affiliations:** 1 Department of Defense Biotechnology High Performance Computing Software Applications Institute, Telemedicine and Advanced Technology Research Center, U.S. Army Medical Research and Materiel Command, Fort Detrick, Maryland, United States of America; 2 J. Craig Venter Institute, Rockville, Maryland, United States of America; 3 Bacteriology Division, U.S. Army Medical Research Institute of Infectious Diseases, Fort Detrick, Maryland, United States of America; Heinrich Heine University, GERMANY

## Abstract

Burkholderia pathogenicity relies on protein virulence factors to control and promote bacterial internalization, survival, and replication within eukaryotic host cells. We recently used yeast two-hybrid (Y2H) screening to identify a small set of novel Burkholderia proteins that were shown to attenuate disease progression in an aerosol infection animal model using the virulent *Burkholderia mallei* ATCC 23344 strain. Here, we performed an extended analysis of primarily nine *B. mallei* virulence factors and their interactions with human proteins to map out how the bacteria can influence and alter host processes and pathways. Specifically, we employed topological analyses to assess the connectivity patterns of targeted host proteins, identify modules of pathogen-interacting host proteins linked to processes promoting infectivity, and evaluate the effect of crosstalk among the identified host protein modules. Overall, our analysis showed that the targeted host proteins generally had a large number of interacting partners and interacted with other host proteins that were also targeted by *B. mallei* proteins. We also introduced a novel Host-Pathogen Interaction Alignment (HPIA) algorithm and used it to explore similarities between host-pathogen interactions of *B. mallei, Yersinia pestis*, and *Salmonella enterica*. We inferred putative roles of *B. mallei* proteins based on the roles of their aligned *Y. pestis* and *S. enterica* partners and showed that up to 73% of the predicted roles matched existing annotations. A key insight into Burkholderia pathogenicity derived from these analyses of Y2H host-pathogen interactions is the identification of eukaryotic-specific targeted cellular mechanisms, including the ubiquitination degradation system and the use of the focal adhesion pathway as a fulcrum for transmitting mechanical forces and regulatory signals. This provides the mechanisms to modulate and adapt the host-cell environment for the successful establishment of host infections and intracellular spread.

## Author Summary

Burkholderia species need to manipulate many host processes and pathways in order to establish a successful intracellular infection in eukaryotic host organisms. *Burkholderia mallei* uses secreted virulence factor proteins as a means to execute host-pathogen interactions and promote pathogenesis. While validated virulence factor proteins have been shown to attenuate infection in animal models, their actual roles in modifying and influencing host processes are not well understood. Here, we used host-pathogen protein-protein interactions derived from yeast two-hybrid screens to study nine known *B. mallei* virulence factors and map out potential virulence mechanisms. From the data, we derived both general and specific insights into Burkholderia host-pathogen infectivity pathways. We showed that *B. mallei* virulence factors tended to target multifunctional host proteins, proteins that interacted with each other, and host proteins with a large number of interacting partners. We also identified similarities between host-pathogen interactions of *B. mallei*, *Yersinia pestis*, and *Salmonella enterica* using a novel host-pathogen interactions alignment algorithm. Importantly, our data are compatible with a framework in which multiple *B. mallei* virulence factors broadly influence key host processes related to ubiquitin-mediated proteolysis and focal adhesion. This provides *B. mallei* the means to modulate and adapt the host-cell environment to advance infection.

## Introduction

*Burkholderia mallei* is the causative agent of glanders, a highly contagious disease that primarily affects horses, mules, and donkeys, but is also transmittable to other mammals through direct contact with infected animals [1]. This host-adapted bacterium is equipped with an extensive set of mechanisms for invasion and modulation of eukaryotic host-cell environments. Key mechanisms of *B. mallei* pathogenicity are encoded in virulence factors (proteins required for virulence) that control and promote pathogenic internalization, survival, and replication within host cells [2, 3]. While a number of *B. mallei* proteins associated with pathogenicity have been characterized and mapped to adhesion, endosomal escape and evasion of host-cell autophagy, actin-based motility, multi-nucleated giant cell formation, replication, and cell-to-cell spread [3–7], the identities of their host targets are largely unknown, and the underlying mechanisms by which the bacterial proteins affect these processes are poorly understood.

In our previous study [8], we used a combined computational and experimental strategy to systematically identify and characterize the interactions between *B. mallei* virulence factors and their host targets. We employed several bioinformatics approaches to identify and select a small number of putative and known virulence factors, and used yeast two-hybrid (Y2H) assays to identify their interacting protein partners in human and murine hosts. The analysis of these host-*B. mallei* protein-protein interactions (PPIs) allowed us to identify three novel *B. mallei* ATCC 23344 virulence factors and show that they attenuated *B. mallei* virulence in mouse aerosol challenge experiments. Although our PPI data contained extensive interactions between multiple host proteins and *B. mallei* proteins, we did not fully explore these data to more generally characterize *B. mallei* virulence mechanisms. Here, we performed a systematic analysis of these interactions to investigate the mechanisms by which *B. mallei* virulence factors interact with host proteins to establish infection, evade host immune responses, and spread within the host. We evaluated whether the virulence factors target specific (non-random) host proteins and processes and whether they jointly affect entry into and survival within the host cells. Furthermore, we evaluated whether we could detect commonalities in Gram-negative bacterial host-pathogen interactions among *B. mallei*, *Yersinia pestis*, and *Salmonella enterica* virulence factors.

A number of studies have used small- or large-scale experiments to analyze Gram-negative bacteria and their host interactions [9–13]. Although the identified interactions represent only a fraction of all possible interactions between host and pathogen proteins (ranging from less than 10 interactions to a few thousand PPIs), they have proved to be a valuable source of information about bacterial pathogenicity mechanisms. Analyses of these host-pathogen PPI datasets showed that virulence-associated pathogen proteins preferentially target host proteins involved in biological processes essential for cell vitality, e.g., signaling, cell cycle, or immune response [9–13]. Additionally, other studies demonstrated that similarities in host-pathogen PPIs can be used to predict novel host proteins that are targeted by bacterial proteins [14–16].

Our analysis showed that *B. mallei* virulence factors targeted host proteins that had a large number of interacting partners and were closely connected to each other. In addition, the analysis revealed specific host processes relevant to *B. mallei* virulence factors’ pathogenicity, e.g., signaling and communication, protein modification and regulation, and cytoskeleton organization, and suggested that virulence factors preferentially targeted multifunctional host proteins, thereby affecting multiple host cellular processes simultaneously. When we used all of our interaction data, including host interactions with putative but not validated *B. mallei* virulence factors, we identified additional host processes and molecular pathways that were previously experimentally associated with *B. mallei* pathogenicity [2, 17–21]. Moreover, our evaluation of the relationship between targeted host proteins involved in different processes and pathways supported a previously observed mechanism for bacterial interference with eukaryotic hosts: virulence factors can focus interference by targeting key host proteins whose effect can propagate through and influence multiple host processes and pathways [2, 17, 18]. Additionally, we introduced a novel Host-Pathogen Interaction Alignment (HPIA) algorithm and used it to explore similarities between host-pathogen interactions of *B. mallei*, *Y. pestis* [13], and *Salmonella enterica* subsp. *enterica* serovar Typhimurium [12]. Using the HPIA algorithm, we identified a statistically significant number of functionally similar host-pathogen interactions between these three PPI datasets. We inferred putative roles for *B. mallei* proteins based on the role of their aligned *Y. pestis* and *S. enterica* partners and showed that up to 73% of the putatively annotated *B. mallei* protein roles matched their existing annotations.

Our findings show that host-pathogen interactions represent a rich source of information about molecular mechanisms of pathogenicity. A key insight from these analyses into Burkholderia pathogenicity is the concerted targeting of the ubiquitination degradation system and use of the focal adhesion pathway as a fulcrum for signaling and changing cell morphology. These mechanisms provide *B. mallei* with the ability to modulate and adapt the host-cell environment to establish intracellular host infections.

## Results/Discussion

**Fig 1:**
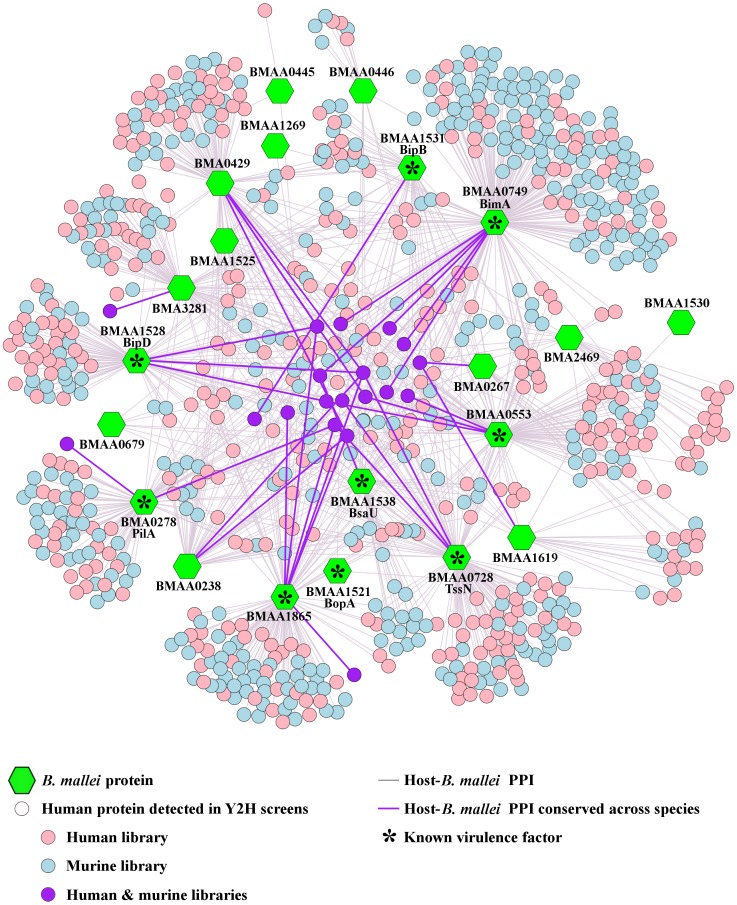
*B. mallei*-human host-pathogen protein interactions. The set of human-*B. mallei* protein-protein interactions (PPIs) was created by merging human-*B. mallei* and orthologous murine-*B. mallei* interaction data. The set consists of 1,235 unique interactions (gray and purple lines) between 21 *B. mallei* (green hexagons) and 828 human proteins (pink, blue, and purple circles). Known virulence factors are also indicated in the graph.

We created an inclusive set of human-*B. mallei* PPIs by merging human-*B. mallei* and orthologous murine-*B. mallei* protein interaction data identified in our previous Y2H screens [8]. The resulting dataset consisted of 1,235 unique interactions between 21 *B. mallei* and 828 human proteins. Fig. 1 shows these interactions and their Y2H-library origins. It also shows that the majority of the *B. mallei* proteins interacted with unique host proteins, i.e., 615 (74%) of host proteins interacted with a single *B. mallei* protein. Importantly, the bulk of the host-*B. mallei* interactions (72% or 890 interactions) involved nine known *B. mallei* virulence factors: PilA, BimA, BopA, BipD, BipB, BsaU, BMAA1865, TssN, and BMAA0553 (Table 1) [22]. These nine *B. mallei* virulence factors interacted with 663 human proteins (80% of all identified host proteins), implying that the captured data were largely reflective of host-pathogen virulence mechanisms. We start our analysis by assessing the characteristics of the host proteins targeted by these virulence factors.

**Table 1:** Known *B*. *mallei* virulence factors that have been shown to attenuate the disease in animal models.

**Protein**	**Secretion System**	**Description**	**Role in Virulence**	**Animal model**	**Ref.**
BMA0278 (PilA)	2	Type IV pilin	Bacterial adhesion	BALB/c	[62]
BMAA0749 (BimA)*	5	Trimeric autotransporter adhesin	Actin-based motility and intracellular/intercellular spread	Murine	[4]
BMAA1521 (BopA)*	3	Secreted effector/translocon	Intracellular survival	BALB/c	[63]
BMAA1528 (BipD)*	3	Transcriptional regulation; Needle tip protein	Invasion of non-phagocytic cells	BALB/c, C57BL/6	[64]
BMAA1531 (BipB)*	3	Secreted effector/translocon	Multi-nucleated giant cell formation	BALB/c	[65]
BMAA1538 (BsaU)*	3	Secretion apparatus; Needle-length control	Bacterial escape from endocytic vesicles	BALB/c	[66]
BMAA1865	3	N/A	Modulation of host ubiquitination; Phagosome escape	BALB/c	[8]
BMAA0728 (TssN)	6	N/A	Interference with host actin cytoskeleton rearrangement	BALB/c	[8]
BMAA0553	2	Ser/Thr protein phosphatase	Interference with host actin cytoskeleton rearrangement	BALB/c	[8]

*Proteins that have been experimentally linked to a particular secretion system.

### Characteristics of host proteins interacting with known *B. mallei* virulence factors

*B. mallei* virulence factors are associated with multiple pathogenic mechanisms of action (Table 1) [3–7], but their direct molecular interactions are not well delineated. First, we applied functional enrichment analyses based on Gene Ontology (GO) annotation data [23] to assess the characteristics of the human proteins targeted by the nine virulence factors. Table 2 shows that these virulence factors interacted with a statistically significant number of human proteins that were associated with *1*) protein ubiquitination and ubiquitin ligase activity, *2*) vesicle organization, and *3*) protein complexes located in the cytoskeleton, in lysosomes, and in the nuclear lumen. These results were consistent with the experimentally observed pathogen interference with host cytoskeleton organization and ubiquitination levels [2, 3, 19–21, 24].

**Table 2:** Enrichment of Gene Ontology (GO) terms for human proteins interacting with *B. mallei* virulence factors.

**Type**	**Term**		**Number of proteins**	***p*-value**	
	**ID**	**Description**		**Original**	**FDR**
GO Biological Processes	GO:0043161	Proteasomal ubiquitin-dependent protein catabolic process	21	4.8∙10^-6^	0.00
	GO:0006457	Protein folding	18	1.2∙10^-4^	0.03
	GO:0000209	Protein polyubiquitination	14	2.0∙10^-4^	0.04
	GO:0016050	Vesicle organization	11	2.5∙10^-4^	0.05
GO Molecular Functions	GO:0004842	Ubiquitin-protein ligase activity	18	3.1∙10^-4^	0.03
	GO:0003729	mRNA binding	10	5.1∙10^-4^	0.05
	GO:0031072	Heat shock protein binding	10	6.3∙10^-4^	0.05
GO Cellular Localizations	GO:0000151	Ubiquitin ligase complex	13	3.7∙10^-4^	0.01
	GO:0015629	Actin cytoskeleton	24	4.8∙10^-4^	0.01
	GO:0030529	Ribonucleoprotein complex	30	7.8∙10^-4^	0.02
	GO:0043220	Schmidt-Lanterman incisure	3	8.5∙10^-4^	0.02
	GO:0034663	Endoplasmic reticulum chaperone complex	2	1.3∙10^-3^	0.03
	GO:0030135	Coated vesicle	19	1.6∙10^-3^	0.03
	GO:0031371	Ubiquitin conjugating enzyme complex	3	2.3∙10^-3^	0.04
	GO:0005764	Lysosome	19	2.5∙10^-3^	0.04
	GO:0032838	Cell projection cytoplasm	4	2.7∙10^-3^	0.04
	GO:0030131	Clathrin adaptor complex	5	3.4∙10^-3^	0.05
	GO:0000803	Sex chromosome	4	3.4∙10^-3^	0.05
	GO:0070971	Endoplasmic reticulum exit site	2	3.8∙10^-3^	0.05
	GO:0031981	Nuclear lumen	80	4.3∙10^-3^	0.05
	GO:0030128	Clathrin coat of endocytic vesicle	3	4.6∙10^-3^	0.05

FDR: False discovery rate calculated using Benjamini and Hochberg multiple test correction [45].

Next, we examined the gross topological properties of the network of interactions formed by the *B. mallei*-targeted host proteins and their interacting host partners, regardless of whether these proteins did or did not interact with the *B. mallei* proteins. We mapped the identified host proteins interacting with *B. mallei* onto a human PPI network [25] consisting of 76,043 physical PPIs among 11,688 proteins. Of the 663 human proteins interacting with the nine *B. mallei* virulence factors, approximately 75% (498) were present in our human PPI network. This set contained proteins that had, on average, a significantly larger number of interacting partners per protein (19.5 vs. 13.0) than would be expected from a corresponding random selection of proteins from the entire human PPI network (Table 3). Among the highest-interacting host proteins targeted by the virulence factors, we found the adapter protein YWHAG (14-3-3 protein gamma) with 376 interactions. This protein, an interacting partner of BimA, has been implicated in the regulation of a large spectrum of signaling pathways [26]. Further topological measures associated with the set of 498 proteins, such as their *clustering coefficient* (a measure of interactions among nearest neighbors), were not different from the random selection (Table 3). We observed small effects on the length of the shortest path between any two proteins in the set, but it was unclear how to associate these topological parameters with *B. mallei* virulence.

**Table 3:** Topological properties of human proteins interacting with *B. mallei*. We evaluated the following properties of the host proteins that interacted with B. mallei proteins based on the human protein-protein interaction (PPI) network [25]: the number of these host proteins in the human PPI network (*N_p_*); the average number of interacting partners (in the human PPI network) of each host protein (*D*); the clustering coefficient, i.e., the number of interactions among the nearest neighbors (*C*); the average shortest path between any two proteins in the set (*SP*); the average number of interacting partners in the human PPI network where both partners interact with *B. mallei* proteins (*D_i_*); and the number of host proteins in the largest connected component ($N_{p}^{LCC}$). The top three rows show the results for the host proteins present in the PPI that interacted with the nine known virulence factors, whereas the three lower rows correspond to host proteins that interacted with all 21 tested *B. mallei* proteins from the yeast two-hybrid screening (known and putative virulence factors). The results for the randomly selected (498 or 619) human proteins from the entire human PPI network (All PPIs) were generated through 10^3^ random repetitions to create averages and standard deviations. The indicated *p*-values correspond to the probability of the observed properties being different from the randomly selected set from all PPIs.

		***N_p_***	***D*** (SD)	***C*** (SD)	***SP*** (SD)	***D_i_*** (SD)	$N_{p}^{LCC}$ (SD)
Known virulence factors	PPIs	498	19.5	0.15	3.41	0.65	202
	All PPIs	498	13.0 (1)	0.17 (0.01)	3.70 (0.04)	0.28 (0.05)	80 (29)
	*p*-value	-	8.8∙10^-8^	0.33	2.0∙10^-11^	1.6∙10^-41^	3.3∙10^-5^
Known and putative virulence factors	PPIs	619	19.5	0.15	3.40	0.85	284
	All PPIs	619	13.0 (1)	0.17 (0.01)	3.70 (0.04)	0.35 (0.06)	136 (35)
	*p*-value	-	4.3∙10^-10^	0.27	1.0∙10^-14^	1.9∙10^-24^	2.2∙10^-5^

SD: standard deviation.

Next, we examined human protein interactions where *both* proteins individually interacted with one or more *B. mallei* proteins. For the 498-protein set, we found 202 unique proteins that participated in 325 human protein-protein interactions. In comparison with randomly selected proteins, the *B. mallei*-targeted proteins were engaged in a significantly larger number of these interactions (0.65 vs. 0.28 on a per-protein basis). A further examination of sets of connected human proteins that also interacted with the virulence factors, revealed the presence of a single, large connected component, i.e., a sub-network in which a path connects any two proteins to each other. This *largest connected component* was composed of 202 proteins and contained the majority (95%) of the 325 interactions between the human proteins interacting with *B. mallei* (Table 3). The other 11 connected components consisted of five or fewer proteins, an observation that was not statistically significant from a random selection of proteins (data not shown). We found a five-fold increase, from 0.28 to 1.53 (0.95*325/202), in the number of human PPIs for each protein in the largest connected component that were all targeted by the virulence factors, compared to a random selection of proteins. These results suggest that a property of the *B. mallei* virulence phenotype is to target well-connected host proteins in a unique set of interconnected host proteins. Next, we used this property to expand on our initial set of GO annotations to better characterize *B. mallei* infectivity and pathogenesis.

### *B. mallei* virulence factors target interactions among host proteins

The analysis of the interactions between the virulence factors and host proteins showed that the targeted human proteins were highly likely to interact among themselves. We hypothesized that interactions among these host proteins are equally important targets as the proteins themselves and could be used to shed light on how virulence factors exert their influence. As detailed in Materials and Methods, we used the largest connected component identified above to detect 93 sets of human PPIs in which, in each set, all human proteins interacted with at least one of the nine known *B. mallei* virulence factors and had the same GO biological process annotations; we denoted these sets *interaction modules*.

**Fig 2:**
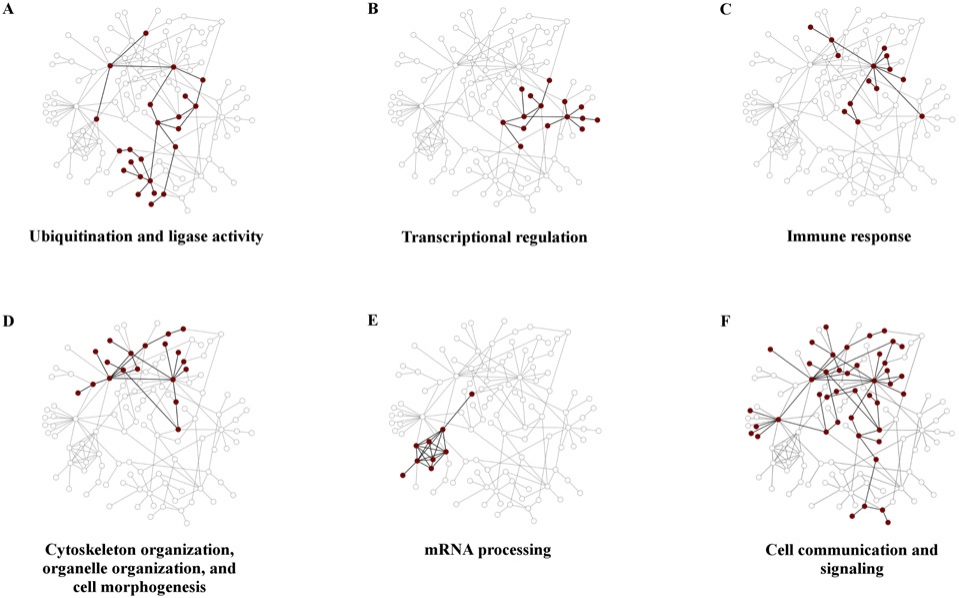
Clustering of human proteins targeted by *B. mallei* known virulence factors. The graphic shows 116 proteins of the largest connected component of the human PPI network that belong to one or more statistically significant interaction modules. Note that each of these human proteins also interacted with one or more known *B. mallei* virulence factors. As exemplified by the annotated interaction modules in A-F, the known virulence factors targeted human proteins that were highly interacting among themselves and belonged to the same biological process. For a list of host proteins that compose each interaction module, see S2 Table.

Table 4 shows that these interaction modules were associated with biological processes related to ligase activity, ubiquitination, protein modification, transcription and translation, immune response, signaling, cytoskeleton organization, development, and mRNA processing. Overall, the identified biological processes were similar to the ones identified when interactions among host proteins were not taken into account; however, they provided an improved annotation granularity. For example, the interaction modules allowed us to identify a biological process termed “positive regulation of protein ubiquitination” instead of just “protein ubiquitination.” Importantly, the analysis provided evidence of a much larger effort to target intracellular host signaling processes, in particular those related to the immune response. Fig. 2 shows the subset of 116 proteins and 163 interactions from the largest connected component that were part of the 93 identified interaction modules and the location of six interaction modules. Each of the interaction modules constituting ubiquitination and ligase activity, transcriptional regulation, immune response, cytoskeleton organization, and mRNA processing, consisted of proteins and interactions that were closely grouped together in the largest connected component (Fig. 2A–E). Fig. 2 also shows that some human proteins are a part of multiple interaction modules, suggesting that *B. mallei* interacts with multifunctional or “moonlighting” host proteins [27]. Multifunctional proteins have been associated with such neurological disorders as Alzheimer’s and Parkinson’s diseases [28], as well as with bacterial virulence in *Helicobacter pylori, Mycobacterium tuberculosis, and Streptococcus pneumonia* [29]. Given the multifaceted role of these proteins in enzymatic catalysis, signal transduction, transcriptional regulation, apoptosis, motility, and growth [30, 31], interactions with them suggest an avenue for *B. mallei* to simultaneously interfere with multiple host-cellular processes to facilitate invasion and survival. In particular, Fig. 2F shows the largest interaction module associated with biological processes linked to multifunctional proteins. This interaction module contained 54 interactions among 44 human proteins associated with various types of regulation (regulation of gene expression, cytokinesis, or apoptosis), signal transduction (GTPase mediated signal transduction and Janus kinase/signal transduction), and response triggering (immune response and response to stress). Additionally, this module contained host-interacting partners of eight out of the nine *B. mallei* virulence factors from our set, lacking only BopA. These results suggest that *B. mallei* virulence factors target multifunctional host proteins to simultaneously interfere with multiple host processes required for normal cellular function.

**Table 4:** Enrichment of Gene Ontology (GO) biological processes in host subnetworks. LCC represents the number of proteins in the largest connected component annotated with a given term; LIM represents the number of proteins in the largest interaction module for a given term; p_GO_ denotes the probability of the same number of proteins as the LCC being annotated with a given GO term solely through a random selection; p_Rp_ denotes the probability that a given number of proteins as the LIM are annotated with a given GO term solely through random selection; p_Rn_ represents the probability that a given number of proteins as the LIM are annotated with a given GO term solely through random selection in a random network that has the same degree distribution as our human network. All *p*-values were assessed using the Benjamini-Hochberg method to meet a maximum false discovery rate threshold of 5% [45]. The table contains only the lowest-level GO terms; the complete data are available in S1 Table.

**Category**	**Term**		**Size**		***p*-value**		
	**ID**	**Description**	**LCC**	**LIM**	**p_GO_**	**p_Rp_**	**p_Rn_**
Protein modification	GO:0051443	Positive regulation of ubiquitin-protein ligase activity	5	3	3.6∙10^-3^	8.2∙10^-3^	4.5∙10^-5^
	GO:0051351	Positive regulation of ligase activity	5	3	4.1∙10^-3^	8.2∙10^-3^	4.5∙10^-5^
	GO:0000209	Protein polyubiquitination	10	8	0.0	0.0	0.0
	GO:0016574	Histone ubiquitination	3	3	5.8∙10^-3^	6.0∙10^-4^	3.7∙10^-7^
	GO:0051248	Negative regulation of protein metabolic process	15	10	7.0∙10^-4^	1.0∙10^-4^	2.0∙10^-4^
Cell cycle	GO:0051320	S phase	6	3	1.0∙10^-2^	1.0∙10^-2^	1.7∙10^-4^
	GO:0051437	Positive regulation of ubiquitin-protein ligase activity involved in mitotic cell cycle	4	3	1.0∙10^-2^	7.4∙10^-3^	2.4∙10^-5^
mRNA processing	GO:0051028	mRNA transport	5	3	9.5∙10^-3^	3.2∙10^-3^	6.4∙10^-5^
	GO:0000398	mRNA splicing, via spliceosome	8	6	7.3∙10^-3^	1.0∙10^-4^	1.0∙10^-6^
Transcription and translation	GO:0006355	Regulation of transcription, DNA-dependent	45	15	7.4∙10^-3^	6.0∙10^-4^	4.3∙10^-4^
	GO:0006413	Translational initiation	8	5	9.0∙10^-4^	0.0	0.0
Signaling and immune response	GO:0007154	Cell communication	71	44	5.7∙10^-3^	0.0	4.4∙10^-5^
	GO:0023052	Signaling	71	44	2.7∙10^-3^	0.0	4.4∙10^-5^
	GO:0035556	Intracellular signal transduction	34	17	7.3∙10^-3^	0.0	1.1∙10^-4^
	GO:0007167	Enzyme linked receptor protein signaling pathway	21	8	1.6∙10^-3^	1.6∙10^-3^	6.8∙10^-4^
	GO:0050852	T cell receptor signaling pathway	7	4	4.0∙10^-4^	1.1∙10^-3^	3.3∙10^-4^
	GO:0016032	Viral reproduction	13	4	2.0∙10^-3^	5.0∙10^-3^	2.1∙10^-2^
	GO:0050688	Regulation of defense response to virus	5	3	1.8∙10^-3^	7.0∙10^-4^	2.0∙10^-4^
	GO:0019221	Cytokine-mediated signaling pathway	12	3	1.8∙10^-3^	1.0∙10^-2^	6.3∙10^-4^
Development	GO:0048731	System development	52	28	1.0∙10^-2^	0.0	6.1∙10^-4^
	GO:0048812	Neuron projection morphogenesis	14	5	1.0∙10^-2^	1.4∙10^-4^	4.7∙10^-4^
Other	GO:0016311	Dephosphorylation	9	4	1.6∙10^-3^	0.0	2.0∙10^-5^
	GO:0006457	Protein folding	8	3	3.6∙10^-3^	3.7∙10^-3^	1.6∙10^-5^
	GO:0016192	Vesicle-mediated transport	20	5	1.0∙10^-2^	3.8∙10^-3^	3.0∙10^-5^
	GO:0007010	Cytoskeleton organization	20	9	3.1∙10^-3^	0.0	4.8∙10^-6^

### Putative *B. mallei* virulence factors improve characterization of *B. mallei targets*

Given that our host-*B. mallei* interaction dataset contained a number of putative virulence factors, we also evaluated the effect of adding these virulence factors into our analysis to characterize host targets. Similarly to the above analyses, we first evaluated the prevalent characteristics of human proteins using GO annotation [23]. The identified molecular annotations largely matched those identified for known virulence factors only, but also included additional GO terms, such as terms related to RNA metabolic processes (S3 Table). Table 3 shows that the analysis of topological properties of host proteins interacting with known and putative virulence factors displayed the same trends observed in the analysis of the interacting partners of known virulence factors. Next, we evaluated the extent to which known and putative virulence factors also targeted connected subsets of host PPIs. We identified 75 statistically significant interaction modules whose GO biological process annotations largely overlapped with the ones identified for interacting partners of known virulence factors only. Although the number of statistically significant interaction modules was smaller than above (an increase in the number of host proteins dilutes the enrichment), the addition of new host proteins increased the size (in terms of proteins and interactions) of previously identified interaction modules (S4 Table). This suggests that with the increase of protein annotation or with the identification of additional host-*B. mallei* PPIs, we will be able to identify larger and more complete host interaction modules targeted by *B. mallei* virulence factors.

**Fig 3:**
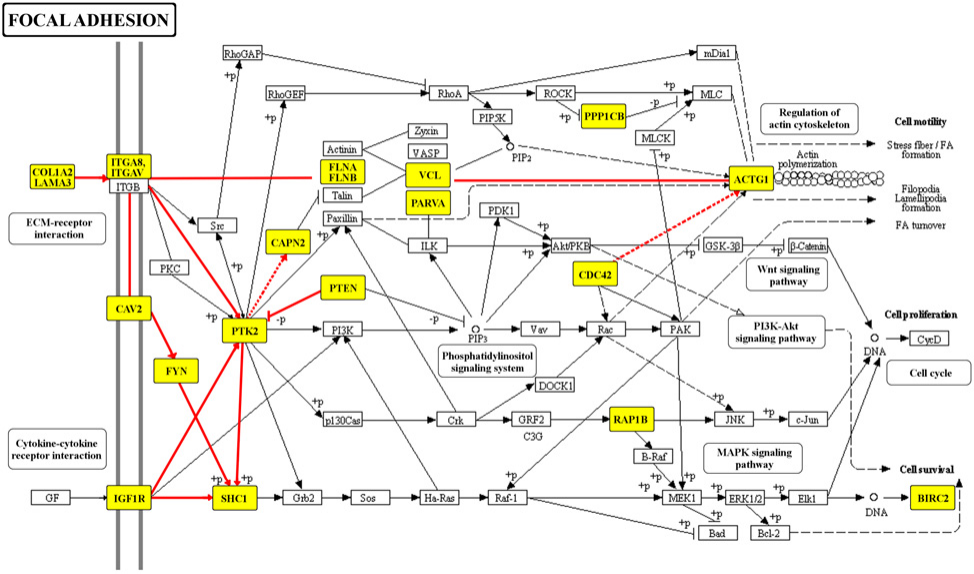
Focal adhesion pathway as a virulence factor target. We identified the Kyoto Encyclopedia of Genes and Genomes (KEGG) focal adhesion pathway as enriched with multiple virulence factor targets. The majority (17 of 20) of host proteins interacting with *B. mallei* virulence factors in this pathway belong to a connected sub-pathway of proteins (yellow boxes and red lines), mainly grouped at the beginning of the pathway. This observation implied that receptors and signaling molecules were likely *B. mallei* targets, corroborating previous observations that pathogens tend to interfere with host processes related to cell communication and actin cytoskeleton organization. The pathway diagram for the focal adhesion pathway was adapted from the KEGG pathway map [32] with permission from the KEGG database administrators.

Consequently, we used all the interactions shown in Fig. 1 to identify biological pathways targeted by all tested *B. mallei* proteins using the Kyoto Encyclopedia of Genes and Genomes (KEGG) annotation database [32]. We identified two statistically significantly enriched host pathways: bacterial invasion of epithelial cells and focal adhesion. Fig. 3 shows that the proteins targeted in the focal adhesion pathway appeared to be coordinated for pathway activation and largely interacted with each other (yellow boxes). The majority of these molecular interactions belonged to a connected sub-pathway located at the beginning of the pathway (the probability of observing such connectivity at random is < 10^-6^), and they provided a link between membrane receptors and signaling events that led to reorganization of the actin cytoskeleton.

### Human-*B. mallei* interactions and their effect on the crosstalk between different biological processes

**Fig 4:**
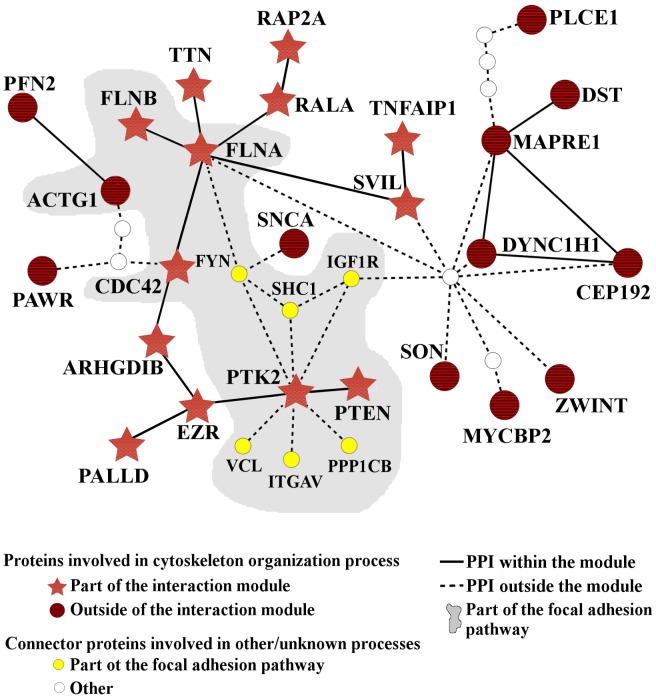
Actin cytoskeleton organization as a virulence factor target. Human proteins targeted by *B. mallei* proteins formed an interaction module that was primarily linked to cytoskeleton organization and focal adhesion. Twenty-five of these proteins were involved in cytoskeleton organization processes; 13 of them (red stars) interacted with each other (forming an interaction module), and the remaining 12 proteins (dark red circles) were on average < 2 nodes (< 3 edges) away from the interaction module. The figure also shows the overlap between the cytoskeleton organization interaction module and the focal adhesion pathway (shaded area), where connecting protein interactions from focal adhesion pathway proteins or other proteins appear as smaller circles and dashed lines. Note that all human proteins shown interacted with one or more *B. mallei* proteins.

One of the most prominently recurring results across all of our analyses was the link between *B. mallei* pathogenicity and host cytoskeleton organization. It has been shown that a number of bacterial pathogens, including *Yersinia*, *Salmonella*, *Shigella*, *Listeria*, and *Burkholderia*, interfere with host signaling pathways to stimulate the host’s cytoskeleton rearrangement [2, 33]. These changes in signaling lead to changes in the host-cell shape and facilitate bacterial internalization and cell-to-cell spread [33]. Fig. 4 shows host proteins that interacted with known and putative *B. mallei* virulence factors that can be directly associated with cytoskeleton organization. The largest statistically significant interaction module, represented by red stars, contained proteins previously identified as bacterial targets vital for host actin cytoskeleton rearrangement, e.g., membrane-associated small GTPases (CDC42 and RALA), Filamin-A (FILA), and Rho GDP-dissociation inhibitor (ARHGDIB) [2, 33]. The remaining cytoskeleton-related host proteins, represented as dark red circles, participated in smaller cytoskeleton organization interaction modules that were, on average, less than two proteins (three interactions or edges) away from the largest module.

**Fig 5:**
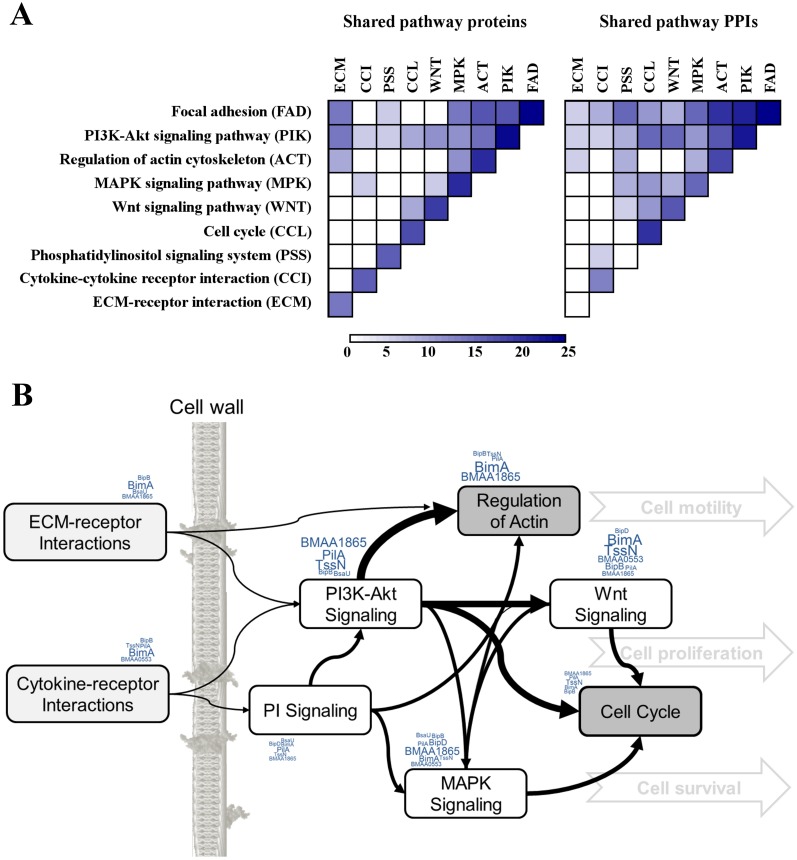
Crosstalk between host pathways targeted by *B. mallei* virulence factors. A) The number of shared proteins and shared pathway protein-protein interactions (PPIs) among human proteins interacting with *B. mallei* that appeared in the focal adhesion pathway and in up to eight other molecular pathways that shared proteins (partially overlapped) with this pathway. The number of shared proteins across pathways was smaller than the number of shared pathway PPIs. B) The location and number of crosstalk interactions affected by *B. mallei* centered around the focal adhesion pathway and appear as arrows with line thicknesses proportional to the number of shared PPIs. The identity and number of virulence factors that target each pathway are illustrated using a word cloud. By preferentially targeting signaling pathways, the effect of one protein modulated through interaction with a virulence factor could propagate and disproportionally influence a larger number of biological processes.

Given that connector proteins (represented as white and yellow circles in Fig. 4) between the cytoskeleton organization interaction modules were annotated with biological processes different from cytoskeleton organization, as well as the multifunctional nature of some cytoskeletal reorganization proteins, we examined the occurrence of shared proteins that interacted with multiple pathways, i.e., pathway crosstalk. Initially, out of all human proteins interacting with the examined *B. mallei* proteins, we evaluated the relationships among proteins involved in the focal adhesion pathway that participated in or interacted with human proteins associated with cytoskeleton organization. The shaded area in Fig. 4 shows that six of 12 proteins were present in both systems. Fig. 5 shows an extension of this analysis that includes *B. mallei* interacting host proteins that are components of eight other molecular pathways that shared proteins with the focal adhesion pathway. Fig. 5A (left) shows that among these nine pathways, the number of pathways that shared one or more proteins was low. However, the number of PPIs connecting the proteins from one pathway with proteins from another pathway was markedly higher [Fig. 5A (right)]. The large number of signaling pathways affected via the focal adhesion pathway magnified the effects of these cross-pathway interactions. Fig. 5B illustrates the propagation and number of cross-pathway interactions that were mediated via the focal adhesion pathway and shows the known virulence factors (Table 1) that can be associated with each pathway. Thus, virulence factors affected biological processes and molecular pathways associated with multiple interconnecting host processes, providing an explanation of how interference with the function of a single protein propagated to and influenced multiple host processes and pathways.

### Using multiple host-pathogen interaction networks to predict the role of pathogen proteins

Our statistical analyses show that the aggregated host-pathogen interaction data could identify host molecular mechanisms targeted by *B. mallei*. However, detecting specific mechanisms of action for *each* pathogen protein based on enrichment analysis of large-scale Y2H protein interaction data is not trivial. This partly stems from experimental, biological, and statistical considerations. For example, the Y2H methodology is biased for certain types of interactions in a non-native environment [34], binding events may or may not be biologically relevant, and statistical testing is hampered by small effect sizes and small statistical power. Conversely, this and previous studies have shown that multiple pathogens tend to target the same host proteins, biological processes, and pathways [2, 9, 10, 18]. Hence, one could potentially use common pathogenic mechanisms to more robustly characterize bacterial proteins and their host targets.

We explored a focused set of human-pathogen interactions derived from putative virulence factor proteins from *S. enterica* and *Y. pestis*; the majority of these proteins are associated with a Type 3 Secretion System (T3SS). These datasets contained 62 host-pathogen interactions for 21 *S. enterica* proteins [12] and 223 interactions for 69 *Y. pestis* proteins [13]. An initial orthology-based approach to retrieve annotations proved too restrictive and did not generate any novel insights into *B. mallei* virulence. Instead, we used an alternative network alignment-based methodology optimized for inter-species alignment, i.e., we differentiated between host and pathogen proteins and avoided mapping host proteins to pathogen proteins and vice versa. As detailed in Materials and Methods, we introduced a novel alignment algorithm (HPIA) designed specifically for the alignment of cross-species interactions. We used the HPIA algorithm to identify similarities between host-pathogen interactions using the *B. mallei*, *S. enterica*, and *Y. pestis* PPI datasets, based on a combined similarity measure that included topological similarity, sequence similarity, and functional similarity. Table 5 lists the *B. mallei* proteins, their aligned protein partners, and inferred function(s) derived from the alignment.

**Table 5:** Putative role of *B. mallei* proteins inferred from host-pathogen interaction network alignment results.

***B. mallei* proteins**				**Proteins aligned to a given *B. mallei* protein**		***Putative* (alignment-predicted) role of a given *B. mallei* protein**
**Locus tag (name)**	**Secretion system association**	**Description**	**Known role**	***S. enterica***	***Y. pestis***	
BMA0278 (PilA)^†^	Type 2	Type IV pilin	Cell adhesion	AvrA	FimA6	- Cell adhesion - Promotion of bacterial survival
BMAA0445 (VgrG)^‡^, *	Type 6	Rhs element Vgr protein	Promoting bacterial survival and replication	SifA	YPO2940	- Cell adhesion - Promotion of bacterial survival
BMAA0446 (VgrG)^‡^, *	Type 6	Rhs element Vgr protein	Promoting bacterial survival and replication	SseG	PilF	- Replication niche establishment
BMAA0749 (BimA)*	Type 5	Hemagglutinin domain protein	Actin based motility	SopB	OmpA	- Promotion of bacterial survival - Bacterial internalization
BMAA1269 (VgrG)	Type 6	Rhs element Vgr protein	Promoting bacterial survival and replication	SseJ	LcrD	- Regulation of T3SS secretion - Promotion of bacterial survival
BMAA1521 (BopA)^†^, *	Type 3	Effector protein	Bacterial internalization and promoting bacterial survival	SptP	YscK	- Bacterial internalization
BMAA1528 (BipD)^†^, *	Type 3	Translocator protein	Bacterial internalization	SipC	YopT	- Bacterial internalization - Interference with host cytoskeleton
BMAA1530 (BipC)^†^, *	Type 3	Effector protein	Bacterial internalization	SipC	LcrV	- Regulation of T3SS activation - Bacterial internalization - Interference with host cytoskeleton
BMAA1531 (BipB)^†^, *	Type 3	Translocator protein	Bacterial internalization	SipB	YscS	- Bacterial internalization
BMAA1538 (BsaU)^†^, *	Type 3	Type 3 secretion protein (needle assembly)	Bacterial internalization	SopE	YscL	- Bacterial internalization - Interference with host cytoskeleton
BMA0429 (Cmk)	Type 3	Cytidylate kinase	Kinase activity; ATP binding	SpiC	YscN	- Regulation of T3SS secretion
BMAA1525 (BapB)*	Type 3	Type 3 secretion protein	N/A	SspH2	TyeA	- Regulation of T3SS secretion - Interference with host ubiquitination
BMAA1865	Type 3	Hypothetical protein	N/A	SopE2	YopE	- Bacterial internalization - Interference with host cytoskeleton
BMAA0728 (TssN)	Type 6	Hypothetical protein	N/A	SseL	YpkA	- Interference with host signaling - Interference with host ubiquitination
BMA0267	-	Pseudogene	N/A	SipA	YPO4044	- Bacterial internalization
BMAA0553	Type 2	Ser/Thr protein phosphatase	N/A	SseI	YPO2113	- Promotion of bacterial survival
BMAA0679	-	Chemotaxis protein CheC	N/A	SspH1	YscY	- Bacterial internalization - Interference with host ubiquitination
BMA2469 (Tkt)	Type 3	Transketolase	N/A	SipA	YscX	- Bacterial internalization - Interference with host cytoskeleton
BMA3281 (FliF)	Type 3	Flagellar M-ring protein	N/A	PipB2	YPMT1.42ac	- Promotion of bacterial survival
BMAA0238	Type 2	Hypothetical protein	N/A	SlrP	YopN	- Regulation of T3SS secretion - Interference with host ubiquitination
BMAA1619	Type 3	Hypothetical protein	N/A	SpvB	HofG7	- Promotion of bacterial survival

For detailed information about the inferred roles, see S5 Table.

^†^Proteins that matched their existing annotation and secretion system (if known).

^‡^Proteins that matched their existing annotation but not the secretion system.

*Proteins that have been experimentally linked to a particular secretion system.

Table 5 shows *B. mallei* proteins with a known/assumed function in pathogenicity and the corresponding functionality, predicted based on the function of their aligned *Y. pestis* and *S. enterica* partners. We identified similarities between T3SS proteins involved in bacterial internalization for all three pathogens, including the orthologs BipB-SipB and BipC-SipC. Additionally, the *B. mallei* PilA protein was aligned to *Y. pestis* fimbrial protein FimA6, another cell adhesion protein. Furthermore, although the *S. enterica* and *Y. pestis* interaction datasets included mainly T3SS proteins, the two *B. mallei* Vgr proteins associated with bacterial survival and replication via Type 6 Secretion Systems (T6SS) were aligned to *S. enterica* and *Y. pestis* proteins also known to promote bacterial survival and replication. Thus, while the aligned proteins may have different roles within each pathogen, it is possible that they interact with a similar type of host proteins, causing the alignment algorithm to capture these similarities. Overall, the alignment-based inferred roles for six out of the 11 (55%) annotated *B. mallei* proteins matched their existing annotation and their corresponding secretion system assignment (Table 5, fourth and second columns, respectively). If we only consider matching functionality and ignore the Vgr association to T6SS, the inferred functions matched the existing annotation in eight of 11 (73%) cases.

For the *B. mallei* virulence factors without known functions in pathogenicity, listed in the lower part of Table 5, the functional mappings from *S. enterica* and *Y. pestis* provided an indication of their mechanistic role in virulence. Of special importance were the three novel virulence factors we identified from the Y2H data and experimentally verified in a *B. mallei* ATCC 23344 aerosol mouse infection model: BMAA1865, BMAA0728, and BMAA0553. The HPIA algorithm identified similarities between BMAA1865, *S. enterica* protein SopE2, and *Y. pestis* protein YopE, based on their interactions with host proteins involved in actin-cytoskeleton rearrangement processes. The alignment also identified similarities between *B. mallei* protein BMAA0728 and *S. enterica* protein SseL based on their interactions with host proteins involved in ubiquitination. Furthermore, the HPIA algorithm identified similarities between *B. mallei* protein BMAA0553 and *S. enterica* protein SseI associated with regulation of the host cytoskeleton and inhibition of cell motility. These results imply that the hypothetical protein BMAA1865 has a role in the host actin-cytoskeleton manipulation, that BMAA0728 has a role in (de)ubiquitination, and that serine/threonine phosphatase BMAA0553 has a role in cytoskeleton regulation. These are the same roles we previously proposed for these three proteins based on the literature review of pathogenic mechanisms [8].

The alignment also identified a putative role of another protein of interest from our previous study, cytidylate kinase BMA0429. The host-pathogen PPI data linked this protein to multiple processes related to pathogenicity. We were not able to test its pathogenicity in an animal model, because this protein appeared to be essential. However, the alignment results imply that this protein had a role in the regulation of T3SS secretion, as it is mapped to two T3SS regulators that are more likely to be localized in the bacterial cytoplasm than to be translocated into the infected cell: SpiC in *S. enterica* and YscN in *Y. pestis* [35–38].

### Multiple *B. mallei* virulence factors target eukaryotic-specific host-cell processes

A key insight into the virulence mechanisms that we could derive from the Y2H interactions was that *B. mallei* targeted eukaryotic-specific cellular mechanisms, such as ubiquitination and focal adhesion. Thus, the specific virulence adaptations retained in the evolution of *B. mallei* as an obligate mammalian pathogen include targeting the ubiquitination degradation/signaling system and using the focal adhesion pathway as a fulcrum for transmitting mechanical forces and regulatory signals. This provides the mechanisms to modulate and adapt the host-cell environment for the successful establishment of host infections.

**Fig 6:**
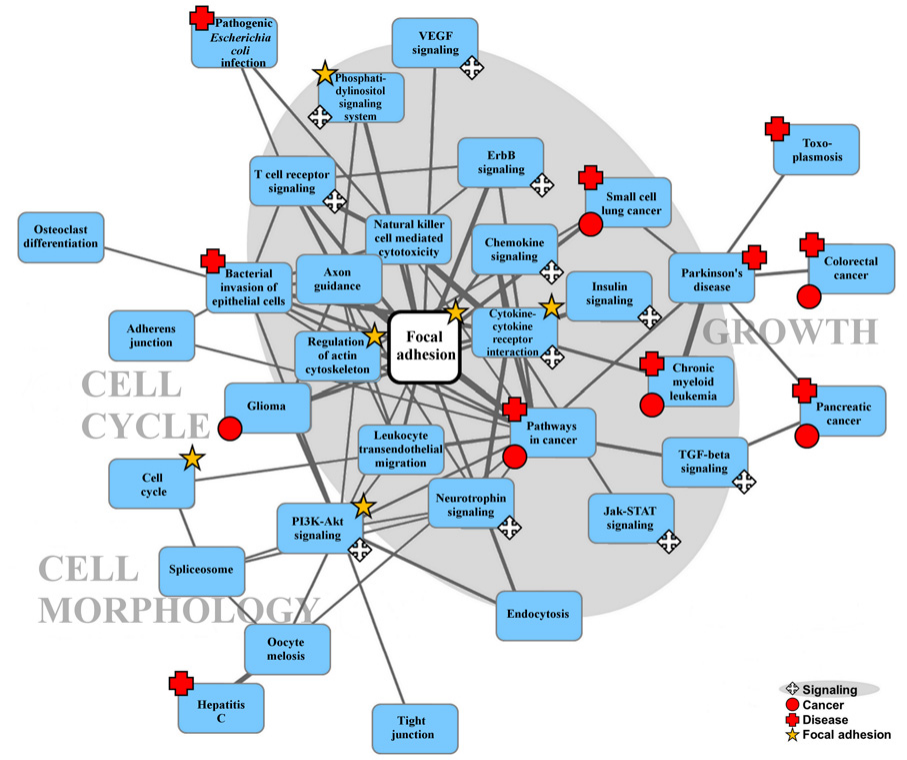
Focal adhesion as a central hub for targeting host cells. The number of shared protein-protein interactions (PPIs) targeted by *B. mallei* virulence factors is shown as lines proportional to the number of PPIs (only connections with 10 or more interactions are illustrated). Physiological, cancer, and disease pathways were all interconnected via signaling pathways that could be affected through the focal adhesion pathway.

Based on our analysis of their host protein interactions and targeted pathways, the nine known virulence factors shared many common points of attack on the host cell’s physiology. Expanding on the cross-talk analysis shown in Fig. 5B, we created an interconnected host-pathway map. We inferred the connection between two pathways from the number of inter-pathway PPIs where both host proteins are interacting with at least one *B. mallei* protein. The larger the number of such host protein pairs, the larger the potential influence *B. mallei* have on the cross-talk between the involved pathways. Fig. 6 shows the extent of this influence between host pathways and the central role the focal adhesion pathway plays in propagating cell signaling and affecting key host cellular processes relevant to *B. mallei* pathogenesis. The pathways implicated in Fig. 5B directly related to focal adhesion are marked with stars in Fig. 6. These, in turn, are interconnected with a large number of signaling pathways (Fig. 6—grey background) that ultimately control cell cycle, morphology, and growth. A number of known disease processes, marked in red symbols, are also interconnected or directly connected to this signaling network. We hypothesized that this virulence factor host-pathogen network is central in controlling key cellular mechanisms that allow *B. mallei* to adapt the host cell environment and ensure robust infection.

### Summary

Given the association of the selected pathogen proteins to secretion systems, the underlying Y2H methodology, and our analysis methodology, our detection capabilities were geared to finding host pathways and biological processes targeted by *B. mallei* via virulence factors. The limitation of this approach is that while a host-pathogen protein interaction may occur, as determined via Y2H experimentation, this type of data does not allow us to resolve when, where, or why such interactions are important. Furthermore, even though the strict statistical threshold at a false discovery rate (FDR) ≤ 5% minimizes the chances of identifying random data correlations, it does not test our hypothesis that the pathway is involved in *B. mallei* virulence. Conversely, the strength of our analysis is that the identified host interactions are dominated by known and validated virulence factors, allowing us to create new hypotheses around the biological interpretation of pathway interaction patterns.

Our results showed that host-pathogen PPIs represent a rich source of information about molecular mechanisms of pathogenicity, and that these interactions can be used to identify and characterize host molecular pathways and processes targeted by pathogens. Specifically, our topological analysis of human-*B. mallei* protein interactions showed that known and putative *B. mallei* virulence factors tend to target multifunctional host proteins, host proteins that interact with each other, and host proteins with a large number of interacting partners. Additionally, the analysis identified a number of host processes and pathways relevant to *B. mallei* pathogenicity, many of which have been linked to bacterial pathogenicity in previous experimental studies, e.g., signaling and communication, protein modification and regulation, cytoskeleton organization, and focal adhesion. Furthermore, the topological analysis suggested that *B. mallei* virulence factors target host molecular processes through interference with their direct and indirect host-interacting partners, implying that the process of pathogenic internalization and intracellular survival requires the modulation of multiple host cellular processes.

We further introduced the novel HPIA algorithm that can be used to identify common sets of host-pathogen interactions by aligning (mapping) host-to-host and pathogen-to-pathogen proteins from two interaction datasets. We used the HPIA algorithm to compare human-*B. mallei* interactions to those of human-*Y. pestis* and human-*S. enterica* and identified a statistically significant number of aligned interactions. We also showed that the resulting alignments could be used to predict roles of *B. mallei* proteins based on the roles of their aligned *Y. pestis* and *S. enterica* partners.

Finally, given that nine of 21 proteins in our dataset are known virulence factors, we could hypothesize on why and how *B. mallei* uses these proteins to overcome multiple defense systems and orchestrate a robust infection process in mammalian hosts. Ultimately, the bacterial host-virulence program is derived from a survival strategy developed in the rhizosphere, i.e., in a generally competitive environment containing multiple, diverse species. Using multiple virulence factors to target eukaryotic-specific mechanisms common to eukaryotic rhizosphere species, *B. mallei* broadly influences key processes in ubiquitination and cell signaling to modulate and adapt the host-cell environment for its benefit.

## Materials and Methods

### Human-*B. mallei* protein interaction set

To create a comprehensive set of human-*B. mallei* PPIs, we merged human-*B. mallei* and murine-*B. mallei* PPI datasets identified in [8]. These datasets contained 586 interactions between 409 human and 21 *B. mallei* proteins, and 797 interactions between 574 murine and 25 *B. mallei* proteins; 19 *B. mallei* proteins appeared in both sets, including nine known *B. mallei* virulence factors (Table 1). When creating the merged set, we considered only a subset of murine-*B. mallei* PPIs in which the *B. mallei* proteins also interacted with human proteins and, thus, had shown the ability to bind to human proteins. The merging procedure consisted of four steps. In the first step, we identified *B. mallei* proteins that interacted with both hosts (19 *B. mallei* proteins). Then, we found human orthologs for each of the 419 (73%) murine proteins that interacted with the *B. mallei* proteins identified in step 1. In the third step, we assessed whether the human orthologs constituted unique proteins, i.e., whether they were not a part of the experimentally detected human-*B. mallei* interactions. If not, we added this interaction into the orthologous human-*B. mallei* dataset. The resulting orthologous dataset consisted of 649 interactions between 419 human proteins and 19 *B. mallei* proteins, corresponding to 82% of the murine-*B. mallei* PPIs. Finally, we merged the experimental human-*B. mallei* dataset with the orthologous human-*B. mallei* dataset to create a merged set of human-*B. mallei* PPIs. The resulting merged dataset consisted of 1,235 unique interactions between 21 *B. mallei* and 828 human proteins (S1 Data and S6 Table). Approximately 72% (890) of these represent interactions among the nine *B. mallei* known virulence factors (Table 1) and 663 unique human proteins. All proteins were annotated by their official gene symbols as defined in the HUGO Gene Nomenclature Committee database [39].

We used the National Center for Biotechnology Information HomoloGene database of homologs (http://www.ncbi.nlm.nih.gov/homologene) to identify human-murine orthologs [40].

### Topological properties of human proteins interacting with *B. mallei* in the human PPI network

We calculated the following topological properties for a set of human proteins interacting with *B. mallei*: *1*) the number of human proteins interacting with *B. mallei* proteins (*N_p_*); *2*) the average number of their interacting partners in the human PPI network (*D*); *3*) the clustering coefficient, i.e., the number of interactions among the nearest neighbors (*C*); the average shortest path between any two proteins in the set (*SP*); the average number of interacting partners in the human PPI network where both partners interact with *B. mallei* proteins (*D_i_*); and the number of host proteins in the largest connected component ($N_{p}^{LCC}$). All calculations were performed in R using the igraph package [41]. We evaluated whether the observed values for each of the five properties were statistically significant as follows. From the human interactome, we randomly selected the same number of proteins as the number of proteins interacting with *B. mallei* virulence factors. Next, we calculated each of the five topological properties for this random set of proteins, repeating the procedure 10^3^ times. This procedure yielded 10^3^ values for each property, which followed a Normal distribution (Normality was evaluated using the quantile-quantile plots and the Kolmogorov-Smirnov test [42], where we found that there was not enough evidence in the data to suggest that the distributions were not Normal). Then, for each property, we evaluated a relationship between the observed value for proteins interacting with *B. mallei* and the values obtained for random protein sets using a Z-score. Finally, we computed the *p-*values corresponding to the resulting Z-scores.

### Gene set functional enrichment analyses

We performed GO and KEGG enrichment analyses in R using the Bioconductor packages BioMart and KEGGgraph, respectively [43, 44]. As the universe of human proteins, we used all constituent proteins from the human PPI network. As GO terms are specified at multiple levels of detail, we used a complete GO tree annotation, excluding the root and the top two levels of GO terms. GO annotation was obtained from BioMart [43]. For the KEGG enrichment analysis, as the universe of human proteins, we used the human proteins available in KEGGgraph that participated in at least one KEGG pathway [44]. All obtained *p*-values were assessed using the Benjamini and Hochberg multiple test correction [45]. We retained only annotations that were enriched at an FDR control level of 0.05, i.e., there is a less than 5% chance that the obtained *p*-values are not statistically significant.

We performed two types of enrichment analysis: standard enrichment analysis and network-based enrichment analysis. In the standard enrichment analysis, we computed the probability of observing the number of proteins annotated with a given term using the hypergeometric distribution. In the network-based enrichment analysis, we first identified the largest connected component in the human interactome that consisted of human proteins interacting with at least one *B. mallei* virulence factor (denoted as *LCC*). Then, we counted the number of proteins *n_t_* in the *LCC* that were annotated with a GO biological process term *t*. Additionally, for each term *t*, we identified connected (sub)networks of *LCC* in which all proteins were annotated with *t*; these we termed *interaction modules*. We denoted such interaction modules as *IM_t_* and the number of proteins in these modules as *m_t_*.

We evaluated whether the observed *IM_t_* interaction modules were statistically significant as follows. First, we evaluated whether the observed interacting proteins and their corresponding biological processes in the *LCC* were statistically significant compared to an equal number of random proteins. For each GO term *t*, we counted the number of proteins in the random set that were annotated with *t* (denoted as *r_t_*). Additionally, for each *t*, we identified interaction modules in which all proteins from the random set were annotated with *t*. We denoted the number of proteins in such interaction modules as *s_t_*. We repeated this procedure 10^4^ times. Given the obtained values, we defined the probability *p_t_* of observing *n_t_* proteins annotated with *t* as

$$p_{t}=\frac{1}{10^{4}}\cdot \overset{10^{4}}{\underset{i=1}{\sum }}k_{i},\;\text{where}\;k_{i}=\{\begin{array}{c} 1\\ 0 \end{array}\begin{array}{ll} & \text{if}\;{\left(r_{t}\right)}_{i}\geq n_{t}\\ & \text{otherwise} \end{array},$$ (1)

and the probability *p_rp_* of observing an interaction module *IM_t_*, given a random set of host proteins, as

$$p_{rp}=\frac{1}{10^{4}}\cdot \overset{10^{4}}{\underset{i=1}{\sum }}k_{i},\;\text{where}\;k_{i}=\{\begin{array}{c} 1\\ 0 \end{array}\begin{array}{ll} & \text{if}\;{\left(s_{t}\right)}_{i}\geq m_{t}\\ & \text{otherwise} \end{array}.$$ (2)

Second, we evaluated whether the observed interacting proteins and their corresponding biological processes were statistically significant compared to random interactions. We randomly rewired the human protein interaction network, while preserving the same degree distribution as observed in the original network. Next, we mapped human proteins from the *LCC* on the rewired network and, for each term *t*, identified interaction modules in which all proteins were annotated with *t*. We denoted the number of proteins in such modules as *w_t_*. We repeated this procedure 10^4^ times. Finally, we calculated the probability *p_rw_* of observing an interaction module, *IM_t_*, given a random set of host interactions, as

$$p_{rw}=\frac{1}{10^{4}}\cdot \overset{10^{4}}{\underset{i=1}{\sum }}k_{i},\;\text{where}\;k_{i}=\{\begin{array}{c} 1\\ 0 \end{array}\begin{array}{ll} & \text{if}\;{\left(w_{t}\right)}_{i}\geq m_{t}\\ & \text{otherwise} \end{array}.$$ (3)

IM_t_ interaction modules with *p_t_* ≤ 0.01, *p_rp_* ≤ 0.01, and *p_rw_* ≤ 0.01 contain a statistically significant number of human proteins and interactions among them, and are statistically significantly enriched in a biological process *t*.

### Human-*S. enterica* and human-*Y. pestis* protein interactions set

The human-*S. enterica* subsp. *enterica* serovar Typhimurium dataset consisted of 62 interactions between 21 *S. enterica* virulence-associated proteins and 51 human proteins identified in several small-scale experiments [12]. The majority of *S. enterica* proteins from this set were associated with the bacterial T3SS. The human-*Y. pestis* PPI dataset consisted of a union of 204 interactions identified by Y2H screens and 23 interactions identified in several small-scale experiments [13]. The combined human-*Y. pestis* PPI dataset contained 223 unique interactions between 69 *Y. pestis* virulence-associated proteins and 125 human proteins. The majority of *Y. pestis* proteins were also associated with the bacterial T3SS. For the basic comparison of the human-*B. mallei*, human-*S. enterica*, and human-*Y. pestis* PPI networks’ characteristics, see S7 Table.

### The HPIA algorithm

Network alignment algorithms [46–49] have been used previously to successfully identify both conserved PPIs [50–55] and phylogenetic relationships between species [51–53]. Although the existing network alignment algorithms can be applied to host-pathogen PPIs, these algorithms are not optimized for inter-species alignment, i.e., they cannot differentiate between different types of proteins, such as host and pathogen proteins, and consequently may map host proteins to pathogen proteins and vice versa. The primary motivation for designing a network alignment algorithm was to be able to use previous data and insights from other host-pathogen interaction studies for interpreting our *B. mallei* host interaction data. If conserved network motifs and interaction exist, we can use this information to infer/predict more complex roles for proteins than just transferring sequence-based annotation information [47, 56]. Thus, we designed the HPIA algorithm specifically for the alignment of host-pathogen interactions to augment the sparsely annotated *B. mallei* protein data.

We have taken a number of considerations into account in designing the algorithm based on the nature of the biological problem at hand. First, due to the non-exhaustive nature of Y2H experimentation, the underlying interaction data are not complete [8]. Second, the selected pathogen species are not identical to each other, i.e., proteins and biological processes have evolved differently between the species. Because of this sparse and diverse nature of the pathogen data at hand, we cannot *a priori* expect to obtain satisfactory alignments based on simply mapping human proteins to each other. In this sense, a “perfect” alignment is never attainable, and instead we must rely on approximate alignments with desirable properties, such as biological consistency and sequence similarity. Hence, we developed an alternate approach for aligning bipartite graphs for which one set of nodes (pathogen) are less-well characterized than the other nodes (human).

For the limited number of host-pathogen interactions we have available for comparisons, our algorithm attempts to identify interactions “conserved” on the functional level rather than at the exact protein level. Thus, we can exploit the fact that all three host-pathogen PPI networks contained interactions with human proteins that participated in similar biological processes. In effect, this allowed us to extend the known annotations from the other networks to the previously uncharacterized *B. mallei* virulence factors.

Furthermore, the algorithm guarantees that host proteins will be aligned only to host proteins and that pathogen proteins will be aligned only to pathogen proteins.

**Notation**. Let *G_1_*(*U_1_, V_1_, E_1_*) and *G_2_*(*U_2_, V_2_, E_2_*) be two bipartite graphs (networks), where *U_1_* and *V_1_* are two disjoint sets of nodes in *G_1_*, *U_2_* and *V_2_* are two disjoint sets of nodes in *G_2_*, and *E_1_* and *E_2_* are sets of edges of *G_1_* and *G_2_* such that every edge in *G_1_* connects a node in *U_1_* to one node in *V_1_*, and every edge in *G_2_* connects a node in *U_2_* to one node in *V_2_* (i.e., no two nodes within the same set are adjacent). Without loss of generality, we can assume that |*U_1_*| *<*|*U_2_*| and |*V_1_*| *<*|*V_2_*| (hence, *G_1_ < G_2_*). The HPIA algorithm is a global network alignment algorithm that uniquely matches each node from *U_1_* to exactly one node in *U_2_*, and each node from *V_1_* to exactly one node in *V_2_*. Formally, the alignment of *G_1_* to *G_2_* can be represented as a set of two ordered pairs {(*u_1_, u_2_*), (*v_1_, v_2_*)}, where *u_1_ ϵ U_1_*, *u_2_ ϵ U_2_*, *v_1_ ϵ V_1_*, and *v_2_ ϵ V_2_*, and no two ordered pairs share a node.

For the host-pathogen interaction networks (*G_1_* and *G_2_*), sets *U_1_* and *U_2_* correspond to pathogen proteins, sets *V_1_* and *V_2_* correspond to host proteins, and sets *E_1_* and *E_2_* correspond to interactions between host and pathogen proteins. Thus, our host-pathogen interaction set corresponds to the host-pathogen network, nodes correspond to proteins, and edges correspond to host-pathogen interactions.

**Algorithm description**. The HPIA algorithm is a seed-and-extend algorithm that consists of the following three steps: *1*) pre-processing, *2*) identification of local alignment, and *3*) identification of global alignment. In this context, we referred to “local alignment” as a smaller area of local network similarity between two networks where not all nodes need to be included, and “global” where all nodes from the smaller network must be aligned to nodes from the larger network [28]. Both mappings are 1-to-1, i.e., one node from one network can be aligned to only one node of the other network. In the first step, the algorithm reads in host-pathogen networks and node annotations, and it calculates similarities between nodes based on either the provided annotation (for node sets with available annotation) or the default topological similarity (for node sets without available annotation). This step also includes handling user-specified seed nodes (nodes that should be mapped to each other). The HPIA algorithm allows a user to provide a set of node pairs (seeds) and/or to employ the algorithm’s feature to automatically search for seed pairs (this search is based on the sequence similarity or equivalent protein names). The HPIA algorithm can treat seed pairs in two ways: *1*) as a suggestion for node alignment, i.e., nodes that should be aligned to each other if other alignment constraints are satisfied (see below), or *2*) as a requirement for the alignment, i.e., seed pairs that have to be aligned to each other. The first step also includes initialization of the aligned pairs list as empty.

In the second step, the HPIA algorithm first identifies a pair of seed nodes (*s_1_, s_2_*), where *s_1_ ϵ G_1_* and *s_2_ ϵ G_2_*, based on the node similarity measures from one of the following three sets (in order of preference): *1*) a set of aligned protein pairs in which both proteins are adjacent to at least one unaligned protein, *2*) a set of user-suggested seed pairs, and *3*) a set of unaligned proteins. All three of these sets contain proteins from the host and pathogen sets and, thus, a pair of seed nodes can come from either the host or pathogen sets of proteins. However, if there are seed node candidates from both sets, the HPIA algorithm preferentially selects a pathogen set. Once selected, the seed pair (*s_1_, s_2_*) is added to the list of aligned pairs. Next, the HPIA algorithm expands around the seed nodes by greedily aligning their direct neighbors *s_1i_* and *s_2j_*(*s_1i_ ϵ N*[*s_1_*] and *s_2j_ ϵ N*[*s_1_*]), based on the given node similarity measure (see below). The HPIA algorithm repeats step two while there exists at least one unaligned pair of host proteins or pathogen proteins adjacent to at least one other unaligned protein. When there are no such pairs left, HPIA proceeds to the third step.

In the third step, the HPIA algorithm greedily aligns all of the remaining unaligned pathogen nodes in *G_1_* to unaligned pathogen nodes *G_2_* and all of the remaining unaligned host nodes in *G_1_* to unaligned host nodes *G_2_*, solely based on node similarity measure. Each pair of nodes is aligned one at a time based on the given node similarity (see below); network connectivity information is not taken into account explicitly (only as a part of the node similarity measure). The results of the alignment are lists of aligned nodes and edges and the following alignment statistics: the total number and percentage of aligned nodes, the number and percentage of aligned pathogen and host nodes, and the number and percentage of aligned edges.

The HPIA algorithm allows a user to provide a set of node pairs (seeds) and to use an automatic search for seed pairs (this search is based on the sequence similarity or equivalent protein names) by specifying an optional parameter, “additionalSeeds.” The HPIA algorithm can treat these seed pairs in two ways. If the “relaxSeeds” option is given, the nodes will be aligned to each other only if other alignment constraints are satisfied as detailed below. If the “relaxSeeds” option is not given, the seed pairs are forced to be aligned to each other. We recommend that the “relaxSeeds” parameter be turned on if the “additionalSeeds” parameter is used.

**Fig 7:**
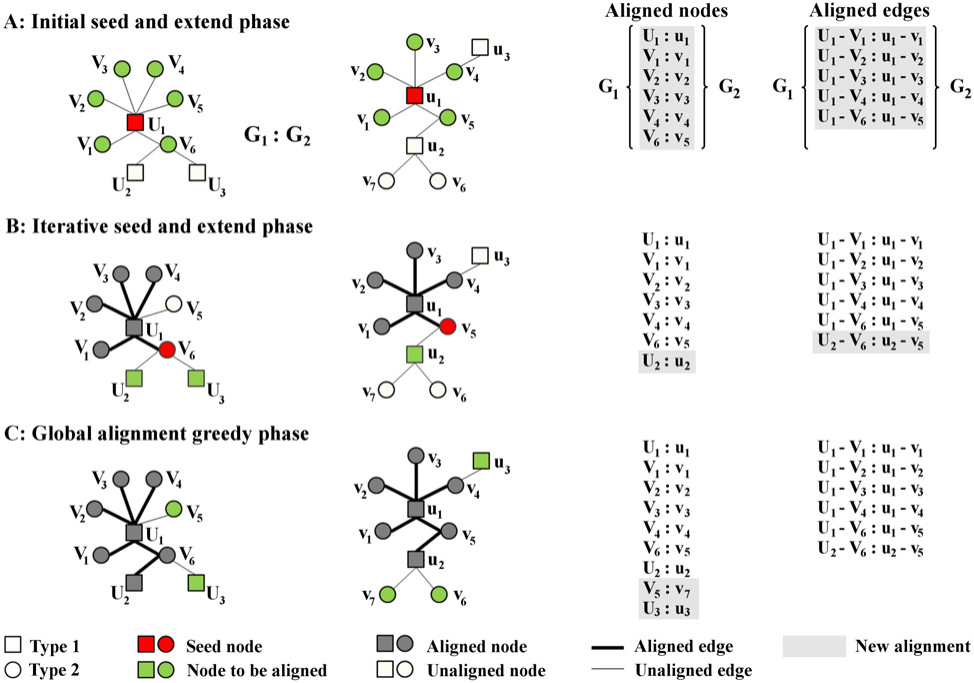
Host-Pathogen Interactions Alignment (HPIA) algorithm. The HPIA algorithm is a seed-and extend algorithm that aligns two bipartite graphs, e.g., two different host-pathogen protein interaction networks. A) Given an initial pair of seed nodes (red nodes U_1_ and u_1_) from two graphs G_1_ (left) and G_2_ (right), the algorithm first aligns seed nodes to each other. Then, it aligns the neighbors of the seed nodes from the first graph (green nodes V_1_-V_6_) to the neighbors of the seed nodes in the second graph (green nodes v_1_-v_5_) based on the node similarity measure (as defined in Equations 6, 8, and 9). This procedure results in six aligned nodes and five aligned edges. B) The algorithm iteratively selects new seeds and extends around them, e.g., it selects nodes V_6_ from G_1_ and v_5_ from G_2_ as new seed nodes and, based on the node similarity measure, aligns their unaligned neighbors U_2_ to u_2_, creating an additional aligned edge (U_2_-V_6_ to u_2_-v_5_). C) When the algorithm cannot find any seed nodes of the same type that have unaligned neighbors, it greedily aligns all of the remaining unaligned nodes based on their type and the node similarity measure. Some nodes may remain unaligned if the graphs’ sizes vary, e.g., when there is no match for v_6_ from G_2_ in G_1_. The HPIA algorithm generates a list of aligned nodes and a list of aligned edges inferred from the aligned nodes.

All ties in the algorithm are broken randomly. The implementation of the HPIA algorithm is also presented as pseudocode in S1 Text. Fig. 7 shows a high-level description of the HPIA alignment algorithm.

**Node similarity measures**. The HPIA algorithm uses one or more topologically and biologically based protein similarity measures to identify conserved interactions. Similarity between two proteins can always be calculated based on at least one of the metrics and, thus, the algorithm always has a metric to match one protein to another. If no node annotation is provided, the HPIA algorithm uses the default topological similarity measure, *S_DT_*(*n_1_, n_2_*), to calculate the similarity between nodes *n_1_ ϵ G_1_* and *n_2_ ϵ G_2_*:

$$S_{DT}(n_{1},n_{2})=\alpha \cdot \frac{\min[\deg(n_{1}),\deg(n_{2})]}{\max[\deg(n_{1}),\deg(n_{2})]}+(1-\alpha )\cdot \frac{\min[nd(n_{1}),nd(n_{2})]}{\max[nd(n_{1}),nd(n_{2})]}$$ (4)

where *deg*(*n*) denotes the degree of node *n* and *nd*(*n*) denotes the neighborhood density of *n*, defined as

$$nd(n)=\sum_{n_{k}\in N[n]}\deg(n_{k})$$ (5)

where *N*[*n*] = *N*(*n*)∪{*n*} represents the closed neighborhood of node *n*, i.e., the node *n* and the set of its adjacent nodes (for a PPI network, this corresponds to a protein and all of its interacting partners). *α* is a parameter in [0, 1] that controls the contribution of the degree of a node to its similarity function. We empirically selected *α* = 0.7, as we wanted to weight the number of direct host-pathogen interactions higher than the number of host-pathogen-host or pathogen-host-pathogen interactions.

If node annotation is provided, the HPIA algorithm defines a similarity between pathogen proteins *u_1_ ϵ U_1_* and *u_2_ ϵ U_2_* as

$$S\left(u_{1},u_{2}\right)=S_{GDV-P}\left(u_{1},u_{2}\right)+S_{EB}\left(u_{1},u_{2}\right)+S_{GO}\left(u_{1},u_{2}\right)$$ (6)

and a similarity between host proteins *v_1_ ϵ V_1_* and *v_2_ ϵ V_2_* as

$$S\left(v_{1},v_{2}\right)=S_{GDV-P}\left(v_{1},v_{2}\right)+S_{GDV-H}\left(v_{1},v_{2}\right)+S_{EB}\left(v_{1},v_{2}\right)+S_{GO}\left(v_{1},v_{2}\right),$$ (7)

where *S_GDV-P_* and *S_GDV-H_* denote the graphlet degree vector similarity [57] derived from the host-pathogen interaction network and the host-PPI network, respectively; *S_EB_* represents the BLAST expected value (E-value) similarity [58], defined as 1– E-value for E-values ≤ 1 and 0 otherwise; and *S_GO_* denotes the GO term annotation similarity calculated using the Jaccard similarity measure [59]. If a specific type of annotation is not provided, the HPIA algorithm assigns the similarity value 0 to the corresponding similarity parameters, e.g., if BLAST E-values are not provided, the value of *S_EB_* for all pairs of nodes is set to 0. We did not add the graphlet degree vector similarity for the pathogen networks because only a few pathogen-PPI networks are available, none of which are for *B. mallei*.

**Data**. For the topological node annotation, we used graphlet degree vectors [57] of all host and pathogen proteins from the host-pathogen PPI networks. Host proteins found in the host PPI network [25] were additionally annotated with another set of graphlet degree vectors calculated based on the host PPI network topology. We used GO annotation [23] downloaded from UNIPROT [60] [the lowest (leaf) level] as the biological node annotation. Additionally, we used BLAST E-values of ≤ 0.01 to define similarities between proteins [58]. Protein sequences were downloaded from UNIPROT and aligned using BLAST pairwise sequence alignment.

**Alignment quality**. To assess the topological quality of the alignment, we used *edge correctness* (EC), defined as the percentage of edges in *G_1_* that were aligned to edges in *G_2_* [51]. To assess the biological quality of the alignment, we evaluated whether the number of aligned protein pairs that share one or more GO term(s) was statistically significant compared to the number we could expect at random using the standard model of sampling without replacement, as described in previous studies [51–53]. We used the same approach to assess the statistical significance of the alignment of two bipartite networks, *G_1_*(*U_1_, V_1_, E_1_*) and *G_2_*(*U_2_, V_2_, E_2_*), with the EC of *x*% (similar to the implementation described above [51–53]). We aligned each pair of host-pathogen interaction networks 30 times and reported the average and standard deviations of the alignment scores over all runs, as well as the best score (S8 Table). We ascertained the robustness of the alignments with respect to the E-value cutoff and observed no significant differences in the results when lowering the cutoff value from 10^-2^ to 10^-3^. To assess the biological quality of the alignment, we evaluated whether the number of aligned protein pairs that share one or more GO term(s) was statistically significant compared to the number we could expect from a random alignment. Given that all obtained alignments were of similar biological quality, we further refined our prediction by using the alignments that had the highest EC score, i.e., we used the alignment with the highest EC score to infer the role of *B. mallei* proteins.

### Implementation and availability

All statistical analyses were performed in R. All networks were plotted using Cytoscape [61]. The cross-species network alignment algorithm was developed in C++. Executable files and examples for the HPIA algorithm are provided at http://www.bhsai.org/downloads/hpia/.

## Supporting Information

S1 Data This file contains a set of human-*B. mallei* PPIs and a set of human PPIs used in this study. (XLSX)

S1 Text This file contains the HPIA algorithm pseudo code and supplementary tables. (DOCX)

S1 Table A list of interaction modules statistically significantly enriched in Gene Ontology (GO) biological processes for human proteins interacting with known *B. mallei* virulence factors. (DOCX)

S2 Table Host proteins associated with distinct biological processes that interacted with known *B. mallei* virulence factors. (DOCX)

S3 Table Gene Ontology (GO) terms and Kyoto Encyclopedia of Genes and Genomes (KEGG) pathways statistically significantly enriched in human proteins interacting with known and/or putative *B. mallei* virulence factors. (DOCX)

S4 Table Subnetworks statistically significantly enriched in Gene Ontology (GO) biological processes for human proteins interacting with known and/or putative *B. mallei* virulence factors. (DOCX)

S5 Table Functional annotation of *B. mallei* proteins inferred from the host-pathogen interaction network alignment. (DOCX)

S6 Table A summary of host-*B. mallei* interactions used in the study. (DOCX)

S7 Table Characteristics of host-pathogen networks used in network alignment. (DOCX)

S8 Table Functional annotation of *B. mallei* proteins inferred from the host-pathogen network alignment. (DOCX)
