# Supplementary material for: Mining Host-Pathogen Protein Interactions to Characterize Burkholderia mallei Infectivity Mechanisms
Source: PLoS Comput Biol. 2015 Mar 4;11(3):e1004088. doi: 10.1371/journal.pcbi.1004088 (PMC4349708; doi:10.1371/journal.pcbi.1004088)
Supplement: S1 Text — (DOCX) [file pcbi.1004088.s002.docx]

**S1 Text:**

**Mining host-pathogen protein interactions to characterize *Burkholderia mallei* infectivity mechanisms**

Vesna Memišević,^1^ Nela Zavaljevski,^1^ Seesandra V. Rajagopala,^2^ Keehwan Kwon,^2^ Rembert Pieper,^2^ David DeShazer,^3^ Jaques Reifman,^1^* and Anders Wallqvist^1^

^1^Department of Defense Biotechnology High Performance Computing Software Applications Institute, Telemedicine and Advanced Technology Research Center, U.S. Army Medical Research and Materiel Command, Fort Detrick, MD

^2^J. Craig Venter Institute, Rockville, MD

^3^Bacteriology Division, U.S. Army Medical Research Institute of Infectious Diseases, Fort Detrick, MD

*Correspondence should be addressed to Jaques Reifman, phone: +1 301 619 7915; fax: +1 301 619 1983; e-mail: jaques.reifman.civ@mail.mil

**HPIA ALGORITHM – PSEUDO CODE**

| **Algorithm 1:** HPIA(G_1_, G_2_)  Align(G_1_, G_2_), where G_1_(U_1_, V_1_, E_1_) and G_2_(U_2_, V_2_, E_2_), u_i_ ϵ U_1_, u_j_ ϵ U_2_, v_k_ ϵ V_1_, and v_l_ ϵ V_2_. A[U_1_, V_1_] = [U_2_, V_2_] is the alignment of nodes from G_1_ to nodes from G_2_. | |
| --- | --- |
| 1  2  3  4  5  6  7  8  9  10  11  12  13  14  15  16  17  18  19  20  21  22  23  24 | Set seed flags to 0: relaxSeeds ←0, additionalSeeds ←0;  Initialize the alignment: A←Ø;  Initialize seeds: S←Ø;  Read networks G_1_ and G_2_,  **if** !empty(user specified fields) **do**  Read seeds: S←{(s_m_, s_n_)};  Set flags relaxSeeds and additionalSeeds;  Read annotation for host and pathogen proteins: AN_H_, AN_P_;  **end if**  **if** additionalSeeds!=0 **do**  Search for additional seed pairs (s_ad1_, s_ad2_);  Add additional seeds: S←{(s_ad1_, s_ad2_)};  **end if**  **if** relaxSeeds == 0 **do**  Add pairs of seed nodes (s_m_, s_n_) into the alignment: A[s_m_] = s_n_;  **end if**  Calculate host node similarity: S_H_;  Calculate pathogen node similarity: S_H_;  **while** ∃ a node in G_1_ that is not in A **do**  Local(G_1_, G_2_);  Global(G_1_, G_2_);  **end while**  Return the alignment A;  Return the alignment statistics; |

| **Algorithm 2:** Local(G_1_, G_2_) | |
| --- | --- |
| 1  2  3  4  5  6  7  8  9  10  11  12  13  14  15  16  17 | **while** ∃ an unaligned pair of nodes, (u_i_, u_j_) or (v_k_, v_l_), adjacent to at least one other node **do**  Initialize candidate seeds: CS ←Ø;  **if** ∃ a pair of nodes (u_i_, u_j_) or (v_k_, v_l_) in A that have at least one  unaligned neighbor then **do**  Add that pair in CS;  **else**  **if** (RS == 1) && (S != Ø) **do**  Add unaligned nodes from S in CS;  **else**  Add all unaligned nodes in CS;  **end if**  **end if**  Based on S_P_ or S_H_  find a pair of proteins (p_m_, p_n_), p_m_ ϵ G_1_, p_n_ ϵ G_2_, from  CS with the highest similarity;  **if** (p_m_, p_n_) ∉ A **do**  Align p_m_ to p_n_: A[p_n_] = p_m_;  **end if**  Based on S_P_ or S_H_, align all unaligned neighbors of p_m_ to all unaligned  neighbors of p_n_;  **end while** |

| **Algorithm 3:** Global(G_1_, G_2_) | |
| --- | --- |
| 1  2  3  4  5  6  7  8 | **for** each unaligned node u_i_ ϵ U_1_ that is not in A **do**  Based on S_P_, find an unaligned node u_j_ ϵ U_2_ that has the highest  similarity to the node u_i_ ϵ U_1_;  Align u_i_ to u_j_: A[u_i_] = u_j_;  **end for**  **for** each unaligned node v_k_ ϵ V_1_ that is not in A **do**  Based on S_H_, find an unaligned node v_l_ ϵ V_2_ that has the highest  similarity to the node v_k_ ϵ V_1_;  Align v_k_ to v_l_: A[v_k_] = v_l_;  **end for** |
