# Supplementary material for: Mining Host-Pathogen Protein Interactions to Characterize Burkholderia mallei Infectivity Mechanisms
Source: PLoS Comput Biol. 2015 Mar 4;11(3):e1004088. doi: 10.1371/journal.pcbi.1004088 (PMC4349708; doi:10.1371/journal.pcbi.1004088)
Supplement: S1 Table — (DOCX) [file pcbi.1004088.s003.docx]

**S1 Table: A list of interaction modules statistically significantly enriched in Gene Ontology (GO) biological processes for human proteins interacting with known *B. mallei* virulence factors.**

| **Category** | **Term** | | **Size** | | ***p*-value** | | |
| --- | --- | --- | --- | --- | --- | --- | --- |
|  | **ID** | **Description** | **LCC** | **LIM** | **p_GO_** | **p_Rp_** | **p_Rn_** |
| Ligase activity | GO:0051443 | Positive regulation of ubiquitin-protein ligase activity | 5 | 3 | 3.6∙10^-3^ | 8.2∙10^-3^ | 4.5∙10^-5^ |
|  | GO:0051438 | Regulation of ubiquitin-protein ligase activity | 5 | 3 | 8.9∙10^-3^ | 9.2∙10^-3^ | 4.5∙10^-5^ |
|  | GO:0051340 | Regulation of ligase activity | 5 | 3 | 9.5∙10^-3^ | 9.2∙10^-3^ | 4.5∙10^-5^ |
|  | GO:0051351 | Positive regulation of ligase activity | 5 | 3 | 4.1∙10^-3^ | 8.2∙10^-3^ | 4.5∙10^-5^ |
| Ubiquitination | GO:0000209 | Protein polyubiquitination | 10 | 8 | 0.0 | 0.0 | 0.0 |
|  | GO:0016567 | Protein ubiquitination | 24 | 16 | 0.0 | 0.0 | 0.0 |
|  | GO:0006511 | Ubiquitin-dependent protein catabolic process | 24 | 8 | 0.0 | 2.0∙10^-4^ | 1.1∙10^-5^ |
|  | GO:0031398 | Positive regulation of protein ubiquitination | 8 | 4 | 6.0∙10^-3^ | 2.3∙10^-3^ | 2.1∙10^-5^ |
|  | GO:0031396 | Regulation of protein ubiquitination | 8 | 4 | 2.1∙10^-3^ | 3.4∙10^-3^ | 2.1∙10^-5^ |
| Histone modification | GO:0016574 | Histone ubiquitination | 3 | 3 | 5.8∙10^-3^ | 6.0∙10^-4^ | 3.7∙10^-7^ |
|  | GO:0016569 | Covalent chromatin modification | 11 | 3 | 1.2∙10^-3^ | 1.0∙10^-2^ | 9.9∙10^-5^ |
|  | GO:0016570 | Histone modification | 11 | 3 | 1.1∙10^-3^ | 1.0∙10^-2^ | 3.7∙10^-7^ |
| Protein modification | GO:0018193 | Peptidyl-amino acid modification | 16 | 5 | 4.3∙10^-3^ | 3.0∙10^-3^ | 4.0∙10^-4^ |
|  | GO:0006464 | Cellular protein modification process | 60 | 41 | 0.0 | 0.0 | 1.5∙10^-6^ |
|  | GO:0070647 | Protein modification by small protein conjugation or removal | 29 | 19 | 0.0 | 0.0 | 0.0 |
|  | GO:0032446 | Protein modification by small protein conjugation | 25 | 17 | 0.0 | 0.0 | 0.0 |
|  | GO:0031401 | Positive regulation of protein modification process | 19 | 6 | 2.7∙10^-3^ | 4.6∙10^-3^ | 4.3∙10^-4^ |
| Metabolic processes | GO:0051248 | Negative regulation of protein metabolic process | 15 | 10 | 7.0∙10^-4^ | 1.0∙10^-4^ | 2.0∙10^-4^ |
|  | GO:0032270 | Positive regulation of cellular protein metabolic process | 20 | 6 | 3.5∙10^-3^ | 6.0∙10^-3^ | 4.5∙10^-4^ |
|  | GO:0051247 | Positive regulation of protein metabolic process | 21 | 6 | 4.5∙10^-3^ | 6.7∙10^-3^ | 5.9∙10^-4^ |
|  | GO:0031323 | Regulation of cellular metabolic process | 73 | 46 | 1.0∙10^-4^ | 0.0 | 1.9∙10^-4^ |
|  | GO:0060255 | Regulation of macromolecule metabolic process | 72 | 49 | 0.0 | 0.0 | 1.0∙10^-4^ |
|  | GO:0080090 | Regulation of primary metabolic process | 71 | 48 | 2.0∙10^-4^ | 0.0 | 6.7∙10^-5^ |
|  | GO:0006508 | Proteolysis | 27 | 8 | 0.0 | 7.0∙10^-4^ | 1.7∙10^-5^ |
| **Category** | **Term** | | **Size** | | ***p*-value** | | |
|  | **ID** | **Description** | **LCC** | **LIM** | **p_GO_** | **p_Rp_** | **p_Rn_** |
| Catabolic processes | GO:0010498 | Proteasomal protein catabolic process | 15 | 5 | 0.0 | 2.1∙10^-3^ | 5.6∙10^-5^ |
|  | GO:0043161 | Proteasomal ubiquitin-dependent protein catabolic process | 15 | 5 | 0.0 | 2.1∙10^-3^ | 5.6∙10^-5^ |
|  | GO:0019941 | Modification-dependent protein catabolic process | 24 | 8 | 0.0 | 2.0∙10^-4^ | 1.1∙10^-5^ |
|  | GO:0043632 | Modification-dependent macromolecule catabolic process | 24 | 8 | 0.0 | 2.0∙10^-4^ | 1.1∙10^-5^ |
|  | GO:0051603 | Proteolysis involved in cellular protein catabolic process | 24 | 8 | 0.0 | 2.0∙10^-4^ | 1.1∙10^-5^ |
|  | GO:0044265 | Cellular macromolecule catabolic process | 28 | 8 | 0.0 | 8.0∙10^-4^ | 6.7∙10^-5^ |
|  | GO:0044257 | Cellular protein catabolic process | 24 | 8 | 0.0 | 2.0∙10^-4^ | 1.1∙10^-5^ |
|  | GO:0030163 | Protein catabolic process | 28 | 13 | 0.0 | 0.0 | 1.5∙10^-4^ |
|  | GO:0009057 | Macromolecule catabolic process | 32 | 13 | 0.0 | 0.0 | 4.5∙10^-4^ |
|  | GO:0009894 | Regulation of catabolic process | 14 | 5 | 7.3∙10^-3^ | 9.0∙10^-4^ | 6.7∙10^-4^ |
| Cell cycle | GO:0051726 | Regulation of cell cycle | 19 | 7 | 2.5∙10^-3^ | 2.8∙10^-3^ | 2.8∙10^-5^ |
|  | GO:0051320 | S phase | 6 | 3 | 1.0∙10^-2^ | 1.0∙10^-2^ | 1.7∙10^-4^ |
|  | GO:0051437 | Positive regulation of ubiquitin-protein ligase activity involved in mitotic cell cycle | 4 | 3 | 1.0∙10^-2^ | 7.4∙10^-3^ | 2.4∙10^-5^ |
| mRNA processing | GO:0051028 | mRNA transport | 5 | 3 | 9.5∙10^-3^ | 3.2∙10^-3^ | 6.4∙10^-5^ |
|  | GO:0006397 | mRNA processing | 15 | 9 | 2.0∙10^-4^ | 0.0 | 0.0 |
|  | GO:0016071 | mRNA metabolic process | 21 | 9 | 1.0∙10^-4^ | 2.0∙10^-4^ | 7.0∙10^-6^ |
|  | GO:0006396 | RNA processing | 18 | 9 | 1.0∙10^-3^ | 0.0 | 1.8∙10^-6^ |
|  | GO:0008380 | RNA splicing | 14 | 9 | 1.0∙10^-4^ | 0.0 | 7.4∙10^-7^ |
|  | GO:0000398 | mRNA splicing, via spliceosome | 8 | 6 | 7.3∙10^-3^ | 1.0∙10^-4^ | 1.0∙10^-6^ |
|  | GO:0000375 | RNA splicing, via transesterification reactions | 8 | 6 | 7.7∙10^-3^ | 1.0∙10^-4^ | 1.1∙10^-6^ |
|  | GO:0000377 | RNA splicing, via transesterification reactions with bulged adenosine as nucleophile | 8 | 6 | 5.8∙10^-2^ | 1.0∙10^-4^ | 1.1∙10^-6^ |
| Transcriptional regulation | GO:0032774 | RNA biosynthetic process | 50 | 15 | 4.4∙10^-3^ | 1.2∙10^-3^ | 7.5∙10^-4^ |
|  | GO:0051252 | Regulation of RNA metabolic process | 47 | 15 | 4.5∙10^-3^ | 7.0∙10^-4^ | 5.6∙10^-4^ |
|  | GO:2001141 | Regulation of RNA biosynthetic process | 45 | 15 | 8.1∙10^-3^ | 7.0∙10^-4^ | 4.3∙10^-4^ |
|  | GO:0006351 | Transcription, DNA-dependent | 48 | 15 | 5.2∙10^-3^ | 2.0∙10^-4^ | 2.0∙10^-4^ |
|  | GO:0006355 | Regulation of transcription, DNA-dependent | 45 | 15 | 7.4∙10^-3^ | 6.0∙10^-4^ | 4.3∙10^-4^ |
| Translational initiation | GO:0006412 | Translation | 14 | 5 | 1.0∙10^-2^ | 2.0∙10^-4^ | 1.8∙10^-6^ |
|  | GO:0006413 | Translational initiation | 8 | 5 | 9.0∙10^-4^ | 0.0 | 0.0 |
| **Category** | **Term** | | **Size** | | ***p*-value** | | |
|  | **ID** | **Description** | **LCC** | **LIM** | **p_GO_** | **p_Rp_** | **p_Rn_** |
| Signaling | GO:0051716 | Cellular response to stimulus | 76 | 48 | 2.0∙10^-3^ | 0.0 | 5.5∙10^-5^ |
|  | GO:0007165 | Signal transduction | 67 | 43 | 1.4∙10^-3^ | 0.0 | 1.4∙10^-5^ |
|  | GO:0007154 | Cell communication | 71 | 44 | 5.7∙10^-3^ | 0.0 | 4.4∙10^-5^ |
|  | GO:0023052 | Signaling | 71 | 44 | 2.7∙10^-3^ | 0.0 | 4.4∙10^-5^ |
|  | GO:0035556 | Intracellular signal transduction | 34 | 17 | 7.3∙10^-3^ | 0.0 | 1.1∙10^-4^ |
|  | GO:0007166 | Cell surface receptor signaling pathway | 41 | 21 | 4.7∙10^-3^ | 0.0 | 3.0∙10^-4^ |
|  | GO:0007167 | Enzyme linked receptor protein signaling pathway | 21 | 8 | 1.6∙10^-3^ | 1.6∙10^-3^ | 6.8∙10^-4^ |
| Immune response | GO:0002764 | Immune response-regulating signaling pathway | 9 | 5 | 4.3∙10^-3^ | 1.8∙10^-3^ | 2.3∙10^-4^ |
|  | GO:0002253 | Activation of immune response | 9 | 5 | 1.0∙10^-2^ | 1.8∙10^-3^ | 2.3∙10^-4^ |
|  | GO:0002757 | Immune response-activating signal transduction | 9 | 5 | 3.1∙10^-3^ | 1.7∙10^-3^ | 2.3∙10^-4^ |
|  | GO:0050852 | T cell receptor signaling pathway | 7 | 4 | 4.0∙10^-4^ | 1.1∙10^-3^ | 3.3∙10^-4^ |
|  | GO:0050851 | Antigen receptor-mediated signaling pathway | 7 | 4 | 1.7∙10^-3^ | 1.7∙10^-3^ | 3.3∙10^-4^ |
|  | GO:0002429 | Immune response-activating cell surface receptor signaling pathway | 7 | 4 | 2.4∙10^-3^ | 1.7∙10^-3^ | 3.3∙10^-4^ |
|  | GO:0002768 | Immune response-regulating cell surface receptor signaling pathway | 7 | 4 | 3.6∙10^-3^ | 2.1∙10^-3^ | 3.3∙10^-4^ |
|  | GO:0080134 | Regulation of response to stress | 17 | 6 | 1.0∙10^-2^ | 3.2∙10^-3^ | 5.1∙10^-4^ |
|  | GO:0051607 | Defense response to virus | 7 | 4 | 9.0∙10^-2^ | 0.0 | 8.4∙10^-5^ |
|  | GO:0009615 | Response to virus | 9 | 5 | 3.9∙10^-3^ | 0.0 | 1.0∙10^-4^ |
|  | GO:0016032 | Viral reproduction | 13 | 4 | 2.0∙10^-3^ | 5.0∙10^-3^ | 2.1∙10^-2^ |
|  | GO:0043900 | Regulation of multi-organism process | 5 | 3 | 1.0∙10^-2^ | 1.1∙10^-3^ | 2.0∙10^-4^ |
|  | GO:0050688 | Regulation of defense response to virus | 5 | 3 | 1.8∙10^-3^ | 7.0∙10^-4^ | 2.0∙10^-4^ |
|  | GO:0002831 | Regulation of response to biotic stimulus | 5 | 3 | 3.5∙10^-3^ | 8.0∙10^-4^ | 2.0∙10^-4^ |
|  | GO:0071345 | Cellular response to cytokine stimulus | 12 | 3 | 7.1∙10^-3^ | 1.0∙10^-2^ | 6.3∙10^-4^ |
|  | GO:0019221 | Cytokine-mediated signaling pathway | 12 | 3 | 1.8∙10^-3^ | 1.0∙10^-2^ | 6.3∙10^-4^ |
|  | GO:0070887 | Cellular response to chemical stimulus | 29 | 10 | 1.0∙10^-2^ | 7.0∙10^-4^ | 7.0∙10^-4^ |
|  | GO:0071310 | Cellular response to organic substance | 26 | 9 | 2.2∙10^-3^ | 6.0∙10^-4^ | 6.7∙10^-4^ |

| **Category** | **Term** | | **Size** | | ***p*-value** | | |
| --- | --- | --- | --- | --- | --- | --- | --- |
|  | **ID** | **Description** | **LCC** | **LIM** | **p_GO_** | **p_Rp_** | **p_Rn_** |
| Development | GO:0007275 | Multicellular organismal development | 61 | 35 | 2.8∙10^-3^ | 0.0 | 3.3∙10^-4^ |
|  | GO:0048731 | System development | 52 | 28 | 1.0∙10^-2^ | 0.0 | 6.1∙10^-4^ |
|  | GO:0048812 | Neuron projection morphogenesis | 14 | 5 | 1.0∙10^-2^ | 1.4∙10^-4^ | 4.7∙10^-4^ |
|  | GO:0048666 | Neuron development | 17 | 5 | 1.0∙10^-2^ | 3.0∙10^-3^ | 6.0∙10^-4^ |
|  | GO:0031175 | Neuron projection development | 17 | 5 | 5.1∙10^-3^ | 2.6∙10^-3^ | 6.0∙10^-4^ |
|  | GO:0030030 | Cell projection organization | 21 | 11 | 2.4∙10^-3^ | 0.0 | 1.7∙10^-4^ |
|  | GO:0048858 | Cell projection morphogenesis | 15 | 7 | 3.1∙10^-3^ | 0.0 | 6.0∙10^-3^ |
|  | GO:0000904 | Cell morphogenesis involved in differentiation | 18 | 8 | 2.2∙10^-3^ | 0.0 | 5.6∙10^-4^ |
|  | GO:0000902 | Cell morphogenesis | 19 | 9 | 2.0∙10^-3^ | 0.0 | 4.7∙10^-4^ |
|  | GO:0032989 | Cellular component morphogenesis | 20 | 10 | 1.0∙10^-2^ | 1.4∙10^-4^ | 4.6∙10^-3^ |
| Other | GO:0016311 | Dephosphorylation | 9 | 4 | 1.6∙10^-3^ | 0.0 | 2.0∙10^-5^ |
|  | GO:0006457 | Protein folding | 8 | 3 | 3.6∙10^-3^ | 3.7∙10^-3^ | 1.6∙10^-5^ |
|  | GO:0016192 | Vesicle-mediated transport | 20 | 5 | 1.0∙10^-2^ | 3.8∙10^-3^ | 3.0∙10^-5^ |
|  | GO:0010467 | Gene expression | 76 | 47 | 0.0 | 0.0 | 1.2∙10^-4^ |
|  | GO:0006996 | Organelle organization | 41 | 10 | 1.3∙10^-3^ | 1.3∙10^-3^ | 1.0∙10^-4^ |
|  | GO:0007010 | Cytoskeleton organization | 20 | 9 | 3.1∙10^-3^ | 0.0 | 4.8∙10^-6^ |

LCC represents the number of proteins in the largest connected component annotated with a given term; LIM represents the number of proteins in the largest interaction module for a given term; p_GO_ denotes the probability of the same number of proteins as the LCC being annotated with a given GO term solely through a random selection; p_Rp_ denotes the probability that a given number of proteins as the LIM are annotated with a given GO term solely through random selection; p_Rn_ represents the probability that a given number of proteins as the LIM are annotated with a given GO term solely through random selection in a random network that has the same degree distribution as our human network. All *p*-values were assessed using the Benjamini-Hochberg method to meet a maximum false discovery rate threshold of 5% [45].
