# Supplementary material for: Mining Host-Pathogen Protein Interactions to Characterize Burkholderia mallei Infectivity Mechanisms
Source: PLoS Comput Biol. 2015 Mar 4;11(3):e1004088. doi: 10.1371/journal.pcbi.1004088 (PMC4349708; doi:10.1371/journal.pcbi.1004088)
Supplement: S2 Table — (DOCX) [file pcbi.1004088.s004.docx]

**S2 Table: Host proteins associated with distinct biological processes that interacted with known *B. mallei* virulence factors.**

| **Biological process** | **Number of host proteins** | **Host proteins** |
| --- | --- | --- |
|  |  |  |
| Ubiquitination and ligase activity | 25 | PSMA6, CUL1, UBB, SNCA, SMAD4, FBXW7, BIRC2, UBE2E3, UBE2L3, ITCH, UBR5, RNF111, UBE2V2, WDR48, BMI1, RNF2, SIAH1, UFM1, RYBP, MYCBP2, PTEN, CDC42, FLNA, FYN, PCBP2 |
| Transcriptional regulation | 15 | RCOR1, SP1, NFYB, BIRC2, PSMA6, MECP2, SMAD4, ETS1, STAT1, PGR, HIF1A, UBE2L3, ITCH, UBB, PTEN |
| Immune response | 14 | PTPRC, FYN, PLCG1, ITCH, CD247, STAT1, RTN4, PTPN2, SP1, LIFR, FLNB, SNCA, UBB, SLC11A2 |
| Cytoskeleton organization, organelle organization, and cell morphogenesis | 19 | PTPRC, FYN, PLCG1, LIFR, RTN4, CDC42, FLNA, RALA, CD2AP, RAP2A, DNM3, SMAD4, STAT1, TTN, SVIL, KLHL12, ARHGDIB, FLNB, TNFAIP1 |
| mRNA processing | 9 | HNRNPA1, SRSF1, SRSF3, SREK1, QKI, HNRNPF, PCBP2, TARDBP, HNRNPA3 |
| Cell communication and signaling | 44 | ARHGDIB, BCLAF1, BIRC2, CD247, CD2AP, CD48, CDC42, CHUK, CUL1, EPN2, FBXW7, FLNA, FLNB, FYN, G3BP1, IGF1R, ITCH, KLHL12, LIFR, LRP8, LRPAP1, MED14, PLCG1, PPM1B, PPP2CB, PPP2R1A, PRKAA1, PSMA6, PTPN2, PTPRC, QKI, RAB18, RALA, RAP2A, RGS7, RNF138, RTN4, SMAD4, SNCA, STAT1, TJP1, UBB, USP8, YWHAG |

This table corresponds to Figure 2.
