# Supplementary material for: Mining Host-Pathogen Protein Interactions to Characterize Burkholderia mallei Infectivity Mechanisms
Source: PLoS Comput Biol. 2015 Mar 4;11(3):e1004088. doi: 10.1371/journal.pcbi.1004088 (PMC4349708; doi:10.1371/journal.pcbi.1004088)
Supplement: S3 Table — (DOCX) [file pcbi.1004088.s005.docx]

**S3 Table: Gene Ontology (GO) terms and Kyoto Encyclopedia of Genes and Genomes (KEGG)** **pathways statistically significantly enriched in human proteins interacting with known and/or putative *B. mallei* virulence factors.**

| **Type** | **Term** | | **Number of proteins** | ***p*-value** | |
| --- | --- | --- | --- | --- | --- |
|  | **ID** | **Description** |  | **Original** | **FDR** |
| GO biological processes | GO:0006396 | RNA processing | 44 | 3.3∙10^-4^ | 0.05 |
|  | GO:0008380 | RNA splicing | 29 | 4.0∙10^-5^ | 0.01 |
|  | GO:0030433 | ER-associated protein catabolic process | 7 | 3.6∙10^-4^ | 0.05 |
|  | GO:0000209 | Protein polyubiquitination | 16 | 1.8∙10^-4^ | 0.03 |
|  | GO:0031398 | Positive regulation of protein ubiquitination | 15 | 1.0∙10^-4^ | 0.02 |
|  | GO:0006457 | Protein folding | 22 | 2.6∙10^-5^ | 0.01 |
| GO molecular functions | GO:0016874 | Ligase activity | 36 | 3.2∙10^-5^ | 0.01 |
|  | GO:0051082 | Unfolded protein binding | 14 | 1.5∙10^-4^ | 0.02 |
|  | GO:0003729 | mRNA binding | 13 | 3.9∙10^-5^ | 0.01 |
| GO  cellular components | GO:0030125 | Clathrin vesicle coat | 5 | 2.0∙10^-3^ | 0.03 |
|  | GO:0005829 | Cytosol | 133 | 2.2∙10^-8^ | 0.00 |
|  | GO:0030529 | Ribonucleoprotein complex | 39 | 5.6∙10^-5^ | 0.00 |
|  | GO:0030530 | Heterogeneous nuclear ribonucleoprotein complex | 6 | 9.2∙10^-5^ | 0.00 |
|  | GO:0005884 | Actin filament | 8 | 1.6∙10^-3^ | 0.03 |
|  | GO:0043220 | Schmidt-Lanterman incisure | 3 | 1.6∙10^-3^ | 0.03 |
| KEGG pathways | hsa:04510 | Focal adhesion | 20 | 1.1∙10^-5^ | 0.00 |
|  | hsa:05100 | Bacterial invasion of epithelial cells | 11 | 2.0∙10^-4^ | 0.02 |

FDR: False discovery rate calculated using Benjamini and Hochberg multiple test correction [45]
