# Supplementary material for: Mining Host-Pathogen Protein Interactions to Characterize Burkholderia mallei Infectivity Mechanisms
Source: PLoS Comput Biol. 2015 Mar 4;11(3):e1004088. doi: 10.1371/journal.pcbi.1004088 (PMC4349708; doi:10.1371/journal.pcbi.1004088)
Supplement: S4 Table — (DOCX) [file pcbi.1004088.s006.docx]

**S4 Table: Subnetworks statistically significantly enriched in Gene Ontology (GO) biological processes for human proteins interacting with known and/or putative *B. mallei* virulence factors.**

| **Category** | **Term** | | **Size** | | ***p*-value** | | |
| --- | --- | --- | --- | --- | --- | --- | --- |
|  | **ID** | **Description** | **LCC** | **LIM** | **p_GO_** | **p_Rp_** | **p_Rn_** |
| mRNA processing | GO:0016070 | RNA metabolic process | 90 | 59 | 0.0 | 0.0 | 1.8∙10^-6^ |
|  | GO:0006351 | Transcription, DNA-dependent | 67 | 34 | 1.7∙10^-3^ | 0.0 | 1.7∙10^-4^ |
|  | GO:0016071 | mRNA metabolic process | 35 | 18 | 0.0 | 0.0 | 3.0∙10^-6^ |
|  | GO:0006396 | RNA processing | 27 | 14 | 0.0 | 0.0 | 0.0 |
|  | GO:0006397 | mRNA processing | 24 | 14 | 0.0 | 0.0 | 0.0 |
|  | GO:0008380 | RNA splicing | 20 | 14 | 0.0 | 0.0 | 0.0 |
|  | GO:0000375 | RNA splicing, via transesterification reactions | 12 | 9 | 7.0∙10^-4^ | 0.0 | 6.0∙10^-7^ |
|  | GO:0000398 | Nuclear mRNA splicing, via spliceosome | 12 | 9 | 5.0∙10^-4^ | 0.0 | 6.0∙10^-7^ |
|  | GO:0000377 | RNA splicing, via transesterification reactions with bulged adenosine as nucleophile | 12 | 9 | 4∙10^-4^ | 0.0 | 6.0∙10^-7^ |
| Catabolic processes | GO:0030163 | Protein catabolic process | 32 | 14 | 0.0 | 0.0 | 1.6∙10^-4^ |
|  | GO:0044265 | Cellular macromolecule catabolic process | 35 | 9 | 0.0 | 2.0∙10-^3^ | 8.0∙10^-5^ |
|  | GO:0044257 | Cellular protein catabolic process | 28 | 9 | 0.0 | 4.0∙10^-4^ | 1.6∙10^-5^ |
|  | GO:0051603 | Proteolysis involved in cellular protein catabolic process | 28 | 9 | 0.0 | 4.0∙10^-4^ | 1.6∙10^-5^ |
|  | GO:0006508 | Proteolysis | 35 | 9 | 0.0 | 1.2∙10^-3^ | 3.5∙10^-5^ |
|  | GO:0019941 | Modification-dependent protein catabolic process | 28 | 9 | 0.0 | 4.0∙10^-4^ | 1.4∙10^-5^ |
|  | GO:0043632 | Modification-dependent macromolecule catabolic process | 28 | 9 | 0.0 | 4.0∙10^-4^ | 1.4∙10^-5^ |
|  | GO:0006511 | Ubiquitin-dependent protein catabolic process | 28 | 9 | 0.0 | 4.0∙10^-4^ | 1.4∙10^-5^ |
|  | GO:0043161 | Proteasomal ubiquitin-dependent protein catabolic process | 18 | 5 | 0.0 | 4.9∙10^-3^ | 5.2∙10^-5^ |
| Protein modification processes | GO:0006464 | Cellular protein modification process | 75 | 56 | 0.0 | 0.0 | 0.0 |
|  | GO:0070647 | Protein modification by small protein conjugation or removal | 37 | 22 | 0.0 | 0.0 | 0.0 |
|  | GO:0032446 | Protein modification by small protein conjugation | 33 | 20 | 0.0 | 0.0 | 0.0 |
|  | GO:0016567 | Protein ubiquitination | 32 | 19 | 0.0 | 0.0 | 0.0 |
|  | GO:0031401 | Positive regulation of protein modification process | 24 | 10 | 4.0∙10^-3^ | 6.0∙10^-4^ | 1.9∙10^-4^ |
|  | GO:0000209 | Protein polyubiquitination | 10 | 8 | 7.0∙10^-4^ | 0.0 | 0.0 |
|  | GO:0031396 | Regulation of protein ubiquitination | 11 | 4 | 3.0∙10^-4^ | 8.7∙10^-3^ | 6.9∙10^-5^ |
|  | GO:0031398 | Positive regulation of protein ubiquitination | 11 | 4 | 0.0 | 6.8∙10^-3^ | 6.9∙10^-5^ |
| **Category** | **Term** | | **Size** | | ***p*-value** | | |
|  | **ID** | **Description** | **LCC** | **LIM** | **p_GO_** | **p_Rp_** | **p_Rn_** |
| Regulation of phosphorus metabolic processes | GO:0019220 | Regulation of phosphate metabolic process | 33 | 19 | 2.0∙10^-4^ | 0.0 | 1.7∙10^-4^ |
|  | GO:0042325 | Regulation of phosphorylation | 31 | 18 | 4.0∙10^-4^ | 0.0 | 1.9∙10^-4^ |
|  | GO:0001932 | Regulation of protein phosphorylation | 28 | 15 | 1.1∙10^-3^ | 0.0 | 2.4∙10^-4^ |
|  | GO:0045859 | Regulation of protein kinase activity | 23 | 12 | 1.6∙10^-3^ | 0.0 | 2.0∙10^-4^ |
|  | GO:0016311 | Dephosphorylation | 11 | 4 | 2.1∙10^-3^ | 5.0∙10^-4^ | 1.9∙10^-4^ |
| Signaling and communication | GO:0023052 | Signaling | 96 | 66 | 4.0∙10^-3^ | 0.0 | 6.0∙10^-7^ |
|  | GO:0007154 | Cell communication | 98 | 68 | 3.4∙10^-3^ | 0.0 | 3.0∙10^-7^ |
|  | GO:0007165 | Signal transduction | 89 | 61 | 2.8∙10^-3^ | 0.0 | 3.0∙10^-7^ |
|  | GO:0007166 | Cell surface receptor signaling pathway | 55 | 38 | 4.0∙10^-3^ | 0.0 | 1.2∙10^-6^ |
|  | GO:0035556 | Intracellular signal transduction | 46 | 25 | 4.1∙10^-3^ | 0.0 | 5.0∙10^-5^ |
|  | GO:0007167 | Enzyme linked receptor protein signaling pathway | 25 | 18 | 0.1∙10^-2^ | 0.0 | 1.8∙10^-5^ |
|  | GO:0007169 | Transmembrane receptor protein tyrosine kinase signaling pathway | 21 | 12 | 6.9∙10^-3^ | 2.0∙10^-4^ | 1.3∙10^-4^ |
|  | GO:0010627 | Regulation of intracellular protein kinase cascade | 19 | 7 | 6.7∙10^-3^ | 3.2∙10^-3^ | 1.7∙10^-4^ |
|  | GO:0006413 | Translational initiation | 9 | 6 | 1.2∙10^-3^ | 0.0 | 0.0 |
| Regulation of metabolic processes | GO:0051246 | Regulation of protein metabolic process | 46 | 30 | 1.0∙10^-4^ | 0.0 | 1.5∙10^-4^ |
|  | GO:0065009 | Regulation of molecular function | 47 | 29 | 4.8∙10^-3^ | 0.0 | 1.7∙10^-5^ |
|  | GO:0032270 | Positive regulation of cellular protein metabolic process | 25 | 10 | 5.4∙10^-3^ | 8.0∙10^-4^ | 2.1∙10^-4^ |
|  | GO:0009894 | Regulation of catabolic process | 19 | 8 | 2.8∙10^-3^ | 1.0∙10^-4^ | 1.5∙10^-4^ |
|  | GO:0042176 | Regulation of protein catabolic process | 11 | 6 | 1.0∙10^-4^ | 0.0 | 2.2∙10^-4^ |
|  | GO:0010498 | Proteasomal protein catabolic process | 18 | 5 | 0.0 | 5.8∙10^-3^ | 5.2∙10^-5^ |
| Cell death | GO:0012501 | Programmed cell death | 49 | 20 | 1.0∙10^-4^ | 2.0∙10^-4^ | 4.4∙10^-5^ |
|  | GO:0010941 | Regulation of cell death | 41 | 18 | 1.0∙10^-4^ | 0.0 | 2.1∙10^-4^ |
|  | GO:0006915 | Apoptotic process | 46 | 18 | 3.0∙10^-4^ | 4.0∙10^-4^ | 9.0∙10^-5^ |
|  | GO:0043067 | Regulation of programmed cell death | 39 | 17 | 7.0∙10^-4^ | 2.0∙10^-4^ | 7.5∙10^-5^ |
|  | GO:0042981 | Regulation of apoptotic process | 38 | 16 | 1.0∙10^-3^ | 4.0∙10^-4^ | 1.1∙10^-4^ |

| **Category** | **Term** | | **Size** | | ***p*-value** | | |
| --- | --- | --- | --- | --- | --- | --- | --- |
|  | **ID** | **Description** | **LCC** | **LIM** | **p_GO_** | **p_Rp_** | **p_Rn_** |
| Response to stimuli | GO:0051716 | Cellular response to stimulus | 104 | 65 | 1.1∙10^-3^ | 0.0 | 4.9∙10^-5^ |
|  | GO:0009615 | Response to virus | 11 | 7 | 4.1∙10^-3^ | 0.0 | 1.5∙10^-5^ |
|  | GO:0051607 | Defense response to virus | 8 | 4 | 8.0∙10^-4^ | 0.0 | 8.1∙10^-5^ |
|  | GO:0071310 | Cellular response to organic substance | 32 | 17 | 5.9∙10^-3^ | 0.0 | 1.4∙10^-4^ |
| Developmental processes | GO:0007275 | Multicellular organismal development | 86 | 54 | 1.0∙10^-4^ | 0.0 | 4.9∙10^-5^ |
|  | GO:0048856 | Anatomical structure development | 83 | 53 | 1.8∙10^-3^ | 0.0 | 2.7∙10^-5^ |
|  | GO:0048731 | System development | 76 | 46 | 6.0∙10^-4^ | 0.0 | 9.7∙10^-5^ |
|  | GO:0048513 | Organ development | 55 | 23 | 1.0∙10^-3^ | 0.0 | 0.0 |
|  | GO:0032989 | Cellular component morphogenesis | 32 | 18 | 5.5∙10^-3^ | 0.0 | 1.9∙10^-4^ |
|  | GO:0000902 | Cell morphogenesis | 31 | 17 | 0.0 | 0.0 | 2.0∙10^-4^ |
|  | GO:0032990 | Cell part morphogenesis | 26 | 15 | 0.0 | 0.0 | 4.0∙10^-5^ |
|  | GO:0048858 | Cell projection morphogenesis | 26 | 15 | 0.0 | 0.0 | 4.0∙10^-5^ |
|  | GO:0048666 | Neuron development | 29 | 14 | 0.0 | 0.0 | 7.3∙10^-5^ |
|  | GO:0031175 | Neuron projection development | 28 | 13 | 0.0 | 0.0 | 1.3∙10^-4^ |
|  | GO:0048812 | Neuron projection morphogenesis | 25 | 13 | 0.0 | 0.0 | 8.3∙10^-5^ |
|  | GO:0048667 | Cell morphogenesis involved in neuron differentiation | 23 | 12 | 0.0 | 0.0 | 1.3∙10^-4^ |
|  | GO:0007409 | Axonogenesis | 19 | 12 | 1.9∙10^-3^ | 0.0 | 7.7∙10^-5^ |
|  | GO:0022604 | Regulation of cell morphogenesis | 12 | 7 | 6.9∙10^-3^ | 0.0 | 1.2∙10^-5^ |
|  | GO:0010769 | Regulation of cell morphogenesis involved in differentiation | 9 | 4 | 6.3∙10^-3^ | 4.0∙10^-4^ | 4.4∙10^-5^ |
| Other | GO:0016192 | Vesicle-mediated transport | 31 | 9 | 4.0∙10^-4^ | 2.0∙10^-4^ | 5.4∙10^-6^ |
|  | GO:0040011 | Locomotion | 33 | 19 | 6.0∙10^-3^ | 0.0 | 6.3∙10^-5^ |
|  | GO:0007010 | Cytoskeleton organization | 25 | 13 | 4.3∙10^-3^ | 0.0 | 0.0 |
|  | GO:0030030 | Cell projection organization | 32 | 19 | 0.0 | 0.0 | 5.4∙10^-6^ |
|  | GO:0006996 | Organelle organization | 53 | 19 | 1.5∙10^-3^ | 1.0∙10^-4^ | 1.5∙10^-5^ |

LCC represents the number of proteins in the largest connected component annotated with a given term; LIM represents the number of proteins in the largest interaction module for a given term; p_GO_ denotes the probability of the same number of proteins as the LCC being annotated with a given GO term solely through a random selection; p_Rp_ denotes the probability that a given number of proteins as the LIM are annotated with a given GO term solely through random selection; p_Rn_ represents the probability that a given number of proteins as the LIM are annotated with a given GO term solely through random selection in a random network that has the same degree distribution as our human network. All *p*-values were assessed using the Benjamini-Hochberg method to meet a maximum false discovery rate threshold of 5% [45].
