# Supplementary material for: Mining Host-Pathogen Protein Interactions to Characterize Burkholderia mallei Infectivity Mechanisms
Source: PLoS Comput Biol. 2015 Mar 4;11(3):e1004088. doi: 10.1371/journal.pcbi.1004088 (PMC4349708; doi:10.1371/journal.pcbi.1004088)
Supplement: S5 Table — (DOCX) [file pcbi.1004088.s007.docx]

**S5 Table: Functional annotation of *B. mallei* proteins inferred from the host-pathogen interaction network alignment.**

| ***B. mallei* proteins** | | | | **Proteins aligned to a given  *B. mallei* protein** | | **Alignment-based functional annotation of a given *B. mallei* protein** | |
| --- | --- | --- | --- | --- | --- | --- | --- |
| **Locus tag (name)** | **Secretion system association** | **Description** | **Known role** | ***S. enterica* protein** | ***Y. pestis* protein** | **Putative role** | **Evidence** |
| BMA0267 | - | Pseudogene | N/A | SipA (P0CL52) | YPO4044 | - Bacterial internalization | - YPO4044 is a fimbrial protein involved in cell adhesion - SipA is a cell invasion protein that is required for an efficient bacterial internalization. It interferes with host cell actin cytoskeleton |
| BMA0278 (PilA)^†^ | Type 2 | Type IV pilin | Cell adhesion | AvrA | FimA6 | - Cell adhesion. - Promotion of bacterial survival | - Fimbrial protein FimA6 is involved in the cell adhesion process - AvrA is a T3SS effector protein-regulator of *Salmonella*-induced inflammatory response |
| BMA0429 (Cmk) | Type 3 | Cytidylate kinase | Kinase activity; ATP binding | SpiC | YscN | - Regulation of T3SS secretion | - *Y. pestis* studies show that YscN, a protein required for delivery of virulence factors, is more likely to reside at the interface of the T3SS and the bacterial cytoplasm, than to be translocated into the infected cell - *Salmonella* studies show that SpiC is not translocated into the infected cell, but localized in the bacterial cytoplasm and required for secretion of SseB and SseC virulence proteins into infected cells |
| BMA2469 (Tkt) | Type 3 | Transketolase | N/A | SipA (E1WAC6) | YscX | - Bacterial internalization - Interference with host cytoskeleton | - YscX, Yop proteins translocation protein X, is required for Yops and V antigen export - SipA is a cell invasion protein that is required for efficient bacterial internalization; it interferes with the host cell actin cytoskeleton |
| BMA3281 (FliF) | Type 3 | Flagellar  M-ring protein | N/A | PipB2 | YPMT1.42ac | - Promotion of bacterial survival | - Secreted effector protein pipB2 alters host cell physiology and promotes bacterial survival in host tissues |
| BMAA0238 | Type 2 | Hypothetical protein | N/A | SlrP | YopN | - Regulation of T3SS secretion - Interference with host ubiquitination | - Outer membrane protein YopN regulates Yop secretion - Effector protein SlrP alters host cell physiology and promotes bacterial survival in host tissues through interference with the host’s ubiquitination pathway |
| BMAA0445  (VgrG)^‡^* | Type 6* | Rhs element Vgr protein | Promoting bacterial survival and replication | SifA | YPO2940 | - Cell adhesion. - Promotion of bacterial survival | - Fimbrial protein YPO2940 is involved in cell adhesion - Effector protein SifA alters host cell physiology and promotes bacterial survival in host tissues |
| BMAA0446  (VgrG)^‡^* | Type 6* | Rhs element Vgr protein | Promoting bacterial survival and replication | SseG | PilF | - Replication niche establishment | - SseG interacts with phagosomal membranes to establish the replication niche |
| BMAA0553 | Type 2 | Ser/Thr protein phosphatase | N/A | SseI | YPO2113 | - Promotion of bacterial survival | - Secreted effector protein SseI alters host cell physiology and promotes bacterial survival in host tissues |

| ***B. mallei* proteins** | | | | **Proteins aligned to a given  *B. mallei* protein** | | **Alignment based functional annotation of a given *B. mallei* protein** | |
| --- | --- | --- | --- | --- | --- | --- | --- |
| **Locus tag (name)** | **Secretion system association** | **Description** | **Known role** | ***S. enterica* protein** | ***Y. pestis* protein** | **Putative role** | **Evidence** |
| BMAA0749 (BimA)* | Type 5* | Hemagglutinin domain protein | Actin-based motility | SopB | OmpA | - Promotion of bacterial survival Bacterial internalization | - Outer membrane protein A enhances intracellular survival of *Y. pestis* - Inositol phosphate phosphatase SopB is required for invasion and for efficient generation and maintenance of Salmonella-containing vacuoles (SVCs) |
| BMAA1269  (VgrG) | Type 6 | Rhs element Vgr protein | Promoting bacterial survival and replication | SseJ | LcrD | - Regulation of T3SS secretion - Promotion of bacterial survival | - The LcrD protein is responsible for the temperature- and calcium-regulated expression and secretion of proteins involved in virulence - The secreted effector protein SseJ alters host cell physiology and promotes bacterial survival in host tissues |
| BMAA1521 (BopA)^†*^ | Type 3* | Effector protein | Bacterial internalization and promoting bacterial survival | SptP | YscK | - Bacterial internalization | - Yop proteins translocation protein K, YscK, is involved in the translocation of Yop proteins across the bacterial membranes - Secreted effector protein SptP alters host cell physiology and promotes bacterial survival in host tissues |
| BMAA1525 (BapB)^*^ | Type 3* | Type 3 secretion protein | N/A | SspH2 | TyeA | - Regulation of T3SS secretion - Interference with host ubiquitination | - Protein TyeA is involved in the control of Yop release - E3 ubiquitin-protein ligase SspH2 alters host cell physiology and promotes bacterial survival in host tissues, through interference with the host’s ubiquitination pathway |
| BMAA1528 (BipD)^†*^ | Type 3* | Translocator protein | Bacterial internalization | SipC (P0CL47) | YopT | - Bacterial internalization and interference with host cytoskeleton | - Cysteine protease YopT is translocated into infected cells and plays a central role in pathogenesis by cleaving the C-terminus end of the human small GTPase RhoA/ARHA, a regulator of the cytoskeleton - Cell invasion protein SipC is an actin-binding protein that interferes with host cell actin cytoskeleton |
| BMAA1530 (BipC)^†*^ | Type 3* | Effector protein | Bacterial internalization | SipC (E1WAC8) | LcrV | - Regulation of T3SS activation - Bacterial internalization and interference with host cytoskeleton | - Virulence-associated V antigen, LcrV, is possibly involved in calcium regulation of YOP expression - Cell invasion protein SipC is an actin-binding protein that interferes with the host cell actin cytoskeleton |
| BMAA1531 (BipB)^†*^ | Type 3* | Translocator protein | Bacterial internalization | SipB | YscS | - Bacterial internalization | - YscS is a component of the Yop secretion machinery, involved in translocation - Cell invasion protein SipB is required for entry into the host cell through presentation or delivery of SipC at the host cell plasma membrane |
| BMAA1538 (BsaU)^†*^ | Type 3* | Type 3 secretion protein (needle assembly) | Bacterial internalization | SopE | YscL | - Bacterial internalization - Interference with host cytoskeleton | - YscL is a component of the Yop secretion machinery, involved in translocation - SopE acts as a guanine nucleotide exchange factor (GEF). Its activation results in actin cytoskeleton rearrangements and stimulates membrane ruffling, promoting bacterial entry into non-phagocytic cells |

| ***B. mallei* proteins** | | | | | **Proteins aligned to a given  *B. mallei* protein** | | **Alignment based functional annotation of a given *B. mallei* protein** | |
| --- | --- | --- | --- | --- | --- | --- | --- | --- |
| **Locus tag (name)** | | **Secretion system association** | **Description** | **Known role** | ***S. enterica* protein** | ***Y. pestis* protein** | **Putative role** | **Evidence** |
| BMAA0679 | - | | Chemotaxis protein CheC | N/A | SspH1 | YscY | - Bacterial internalization - Interference with host ubiquitination | - Chaperone protein YscY is required for Yops and V antigen export - Effector protein sspH1 alters host cell physiology and promotes bacterial survival in host tissues, through interference with the host’s ubiquitination pathway |
| BMAA0728 (TssN) | Type 6 | | Hypothetical protein | N/A | SseL | YpkA | - Interference with host signaling - Interference with host ubiquitination | - Protein kinase YpkA interferes with host signaling - Deubiquitinase SseL interferes with the host cell ubiquitin pathway by acting as a deubiquitinase in infected host cell |
| BMAA1619 | Type 3 | | Hypothetical protein | N/A | SpvB | HofG7 | - Promotion of bacterial survival | - SpvB is involved in ADP-ribosyltransferase enzymatic activity, promoting bacterial survival in host tissues |
| BMAA1865 | Type 3 | | Hypothetical protein | N/A | SopE2 | YopE | - Bacterial internalization and interference with host cytoskeleton | - Outer membrane virulence protein YopE functions as a GTPase-activating protein, interfering with the host cell actin regulation of Rho GTPases - SopE2 acts as a guanine nucleotide exchange factor (GEF). Its activation results in actin cytoskeleton rearrangements and stimulates membrane ruffling, promoting bacterial entry into non-phagocytic cells |

^†^Proteins that matched their existing annotation and secretion system (if known). ^‡^Proteins that matched their existing annotation but not the secretion system. *Proteins that have been experimentally linked to a particular secretion system.
