# Supplementary material for: Mining Host-Pathogen Protein Interactions to Characterize Burkholderia mallei Infectivity Mechanisms
Source: PLoS Comput Biol. 2015 Mar 4;11(3):e1004088. doi: 10.1371/journal.pcbi.1004088 (PMC4349708; doi:10.1371/journal.pcbi.1004088)
Supplement: S6 Table — (DOCX) [file pcbi.1004088.s008.docx]

**S6 Table: A summary of host-*B. mallei* interactions used in the study.**

| **Locus tag (protein name)** | **Description** | **Number of PPIs detected** | | | **Number of PPIs in the expanded set** |
| --- | --- | --- | --- | --- | --- |
|  |  | **Human** | **Murine** | **Conserved** |  |
| BMA0267 | Pseudogene | 10 | 4 | 1 | 13 |
| BMA0278 (PilA)* | Type IV pilin | 41 | 60 | 2 | 93 |
| BMA0429 (Cmk) | Cytidylate kinase | 59 | 58 | 4 | 104 |
| BMA2469 (Tkt) | Transketolase | 24 | 5 | - | 28 |
| BMA3281 (FliF) | Flagellar M-ring protein | 42 | 43 | 1 | 81 |
| BMAA0238 | Hypothetical protein | 25 | 28 | 2 | 51 |
| BMAA0445 | Rhs element Vgr protein | 2 | 1 | - | 3 |
| BMAA0446 | Rhs element Vgr protein | 7 | 7 | - | 13 |
| BMAA0553* | Ser/Thr protein phosphatase | 59 | 57 | 3 | 110 |
| BMAA0679 | Chemotaxis protein CheC | 7 | - | - | 7 |
| BMAA0728 (TssN)* | Hypothetical protein | 71 | 74 | 3 | 137 |
| BMAA0749 (BimA)* | Hemagglutinin domain protein | 77 | 183 | 5 | 240 |
| BMAA1269 | Rhs element Vgr protein | 1 | 1 | - | 2 |
| BMAA1521 (BopA)* | Effector protein | 1 | 1 | - | 2 |
| BMAA1525 (BapB) | Type 3 secretion protein | 9 | 12 | - | 20 |
| BMAA1528 (BipD)* | Translocator protein | 41 | 49 | 3 | 85 |
| BMAA1530 (BipC) | Effector protein | 1 | - | - | 1 |
| BMAA1531 (BipB)* | Translocator protein | 24 | 23 | 1 | 46 |
| BMAA1538 (BsaU)* | Type 3 secretion protein | 14 | 38 | 1 | 48 |
| BMAA1619 | Hypothetical protein | 14 | 9 | 1 | 22 |
| BMAA1865* | Hypothetical protein | 57 | 84 | 6 | 129 |
| BMA0666 (CysD-1) | Sulfate adenylyltransferase | - | 3 | - | N/A |
| BMAA0729 (TssM)* | TssM protein | - | 2 | - | N/A |
| BMAA1523 (BopE) | Guanine nucleotide exchange factor BopE | - | 27 | - | N/A |
| BMAA1648 | Hypothetical protein | - | 9 | - | N/A |
| BMAA1662 | Response regulator | - | 19 | - | N/A |

* Known virulence factors
