# Supplementary material for: Mining Host-Pathogen Protein Interactions to Characterize Burkholderia mallei Infectivity Mechanisms
Source: PLoS Comput Biol. 2015 Mar 4;11(3):e1004088. doi: 10.1371/journal.pcbi.1004088 (PMC4349708; doi:10.1371/journal.pcbi.1004088)
Supplement: S7 Table — (DOCX) [file pcbi.1004088.s009.docx]

**S7 Table: Characteristics of host-pathogen networks used in network alignment.**

| **Network** | **Secretion system type** | **Number of nodes** | | | **Number of edges** | **Average degree (SD)** | | | **Diameter** |
| --- | --- | --- | --- | --- | --- | --- | --- | --- | --- |
|  |  | **All** | **Host** | **Pathogen** |  | **All** | **Host** | **Pathogen** |  |
| *B. mallei* | T3, T6, O/U | 849 | 828 | 21 | 1,235 | 2.9 (13.0) | 2.4 (2.2) | 119.7 (68.6) | 7 |
| *Y. pestis* | T3, O/U | 194 | 125 | 69 | 223 | 2.3 (2.5) | 4.3 (5.1) | 5.7 (3.6) | 13 |
| *S. enterica* | T3, O/U | 74 | 49 | 25 | 61 | 1.7 (1.3) | 1.6 (0.9) | 3.8 (2.3) | 5 |

T3: Type 3 secretion system; T6: Type 6 secretion system; O/U: Other/unknown; SD: standard deviation
