# Supplementary material for: Mining Host-Pathogen Protein Interactions to Characterize Burkholderia mallei Infectivity Mechanisms
Source: PLoS Comput Biol. 2015 Mar 4;11(3):e1004088. doi: 10.1371/journal.pcbi.1004088 (PMC4349708; doi:10.1371/journal.pcbi.1004088)
Supplement: S8 Table — (DOCX) [file pcbi.1004088.s010.docx]

**S8 Table: Functional annotation of *B. mallei* proteins inferred from the host-pathogen network alignment.**

| **Alignment** | **Protein similarity based on** | **Aligned edges** | | | | **Shared GO terms (best alignment)** | | | | | |
| --- | --- | --- | --- | --- | --- | --- | --- | --- | --- | --- | --- |
|  |  | **Average (SD)** | | **Best** | | **Type** | **1** | **2** | **3** | **4** | **5** |
|  |  | # | % | # | % |  |  |  |  |  |  |
| HBM – HSE | Topology (default) | 53.0 (0.0) | 86.9 (0.0) | 53 | 86.9 | CL | 19 | 5 | - | - | - |
|  |  |  |  |  |  | MF | 2 | - | - | - | - |
|  |  |  |  |  |  | BP | 9 | 2 | - | - | - |
|  | Sequence only | 49.8 (1.0) | 81.7 (1.6) | 51 | 83.6 | CL | 20 | 7* | 3* | 2* | 2* |
|  |  |  |  |  |  | MF | 11 | 5 | - | - | - |
|  |  |  |  |  |  | BP | 16 | 6 | 3* | 2* | 2* |
|  | Multiple factors | 51.6 (1.3) | 84.5 (2.2) | 53 | 86.9 | CL | 35* | 15* | 5* | 2* | 2* |
|  |  |  |  |  |  | MF | 14 | 3 | - | - | - |
|  |  |  |  |  |  | BP | 27* | 14* | 8* | 6* | 6* |
| HBM – HYP | Topology (default) | 91.0 (0.0) | 40.8 (0.0) | 91 | 40.8 | CL | 33 | 4 | - | - | - |
|  |  |  |  |  |  | MF | 3 | 2 | 2 | - | - |
|  |  |  |  |  |  | BP | 11 | 2 | 2 | 2* | 2* |
|  | Sequence only | 89.5 (5.7) | 39.6 (2.5) | 93 | 41.7 | CL | 41* | 11* | 3* | - | - |
|  |  |  |  |  |  | MF | 22 | 6 | - | - | - |
|  |  |  |  |  |  | BP | 23 | 10* | 5* | 3* | 2* |
|  | Multiple factors | 79.3 (3.4) | 35.6 (1.6) | 85 | 38.1 | CL | 100 | 41 | 10 | 3 | 2 |
|  |  |  |  |  |  | MF | 27 | 12 | 3 | - | - |
|  |  |  |  |  |  | BP | 53 | 26 | 15 | 10 | 9 |

HBM – HSE: Human-*B. mallei* – Human-*S. enterica* protein-protein interaction (PPI) alignment; HBM – HYP: Human-*B. mallei* – Human-*Y. pestis* PPI alignment; BP: Gene Ontology (GO) biological process; CL: GO cellular localization; MF: GO molecular function; SD: standard deviation; *statistically significant number of aligned pairs with the same functional annotation (≤ 0.01).

All alignments have a statistically significant number of aligned interactions. Each pair of host-pathogen interaction networks has been aligned 30 times.
